# Functional strain redundancy and persistent phage infection in Swiss hard cheese starter cultures

**DOI:** 10.1038/s41396-021-01071-0

**Published:** 2021-08-06

**Authors:** Vincent Somerville, Hélène Berthoud, Remo S. Schmidt, Hans-Peter Bachmann, Yi Hélène Meng, Pascal Fuchsmann, Ueli von Ah, Philipp Engel

**Affiliations:** 1 Department of Fundamental Microbiology, University of Lausanne, Lausanne, Switzerland; 2 Agroscope, Bern, Switzerland

**Keywords:** Microbial ecology, Applied microbiology

## Abstract

Undefined starter cultures are poorly characterized bacterial communities from environmental origin used in cheese making. They are phenotypically stable and have evolved through domestication by repeated propagation in closed and highly controlled environments over centuries. This makes them interesting for understanding eco-evolutionary dynamics governing microbial communities. While cheese starter cultures are known to be dominated by a few bacterial species, little is known about the composition, functional relevance, and temporal dynamics of strain-level diversity. Here, we applied shotgun metagenomics to an important Swiss cheese starter culture and analyzed historical and experimental samples reflecting 82 years of starter culture propagation. We found that the bacterial community is highly stable and dominated by only a few coexisting strains of *Streptococcus thermophilus* and *Lactobacillus delbrueckii* subsp. *lactis*. Genome sequencing, metabolomics analysis, and co-culturing experiments of 43 isolates show that these strains are functionally redundant, but differ tremendously in their phage resistance potential. Moreover, we identified two highly abundant *Streptococcus* phages that seem to stably coexist in the community without any negative impact on bacterial growth or strain persistence, and despite the presence of a large and diverse repertoire of matching CRISPR spacers. Our findings show that functionally equivalent strains can coexist in domesticated microbial communities and highlight an important role of bacteria-phage interactions that are different from kill-the-winner dynamics.

## Introduction

Natural microbial communities are complex biological systems. They are typically composed of a large number of different species and a high extent of strain-level diversity [1]. Moreover, they can be highly dynamic, influenced by microbial dispersion/migration, variation in physicochemical properties of the environment, nutrient availability, habitat size, interspecies interactions, and phage predation [2]. Therefore, it has remained challenging to track microbial communities in open biological systems and to understand how different factors shape their diversity and eco-evolutionary dynamics.

Microbial communities harnessed by humans for the production of fermented foods are excellent models to investigate these questions because they are typically less complex and grown under more stable conditions than natural communities [3]. Cheese making relies on bacterial starter cultures, which have been propagated in cheese or milk over many generations, and are composed of a few coexisting species [4]. These starter cultures are essential for initiating the cheese making process by degrading proteins and fermenting lactose into lactate, resulting in the acidification and preservation of the milk environment. Two different types of cheese starter cultures (defined and undefined) are being used in today’s cheese-making industry [5]. Defined starter cultures are artificial communities that have been assembled from selected strains based on desired phenotypic properties [6]. In contrast, undefined starter cultures are domesticated communities of unknown composition that were originally isolated from traditional cheese, but since then have been propagated, and shaped, by cycles of freeze drying, reactivation, and growth in milk. One of the major advantages of undefined starter cultures in cheese making is that they are phenotypically more stable and less prone to undergo spontaneous community collapse as frequently observed for defined cultures [7]. The reasons underlying the increased stability are unknown. However, one possibility is that diverse strains or species present in undefined starter cultures make these communities more resilient to environmental stressors [8].

While the abiotic conditions usually remain highly stable when growing the starter culture in milk to produce cheese, bacteriophages are relatively common and pose a risk for the cheese making process [9]. Infection with a virulent phage can cause bacterial lysis, which in turn slows down fermentation and results in a low quality product, or the complete failure of the cheese production [10]. For an undefined Gouda cheese starter culture, which is grown in pasteurized milk at 30 °C (also termed mesophilic starter culture), it has been shown that different strains of *Lactococcus lactis* differ in their susceptibility to phages and that negative frequency dependent selection, in the form of kill-the-winner bacteria-phage interactions, possibly prevent community collapse [11]. High levels of strain diversity may thus promote the functional stability of these communities. On the other hand, evolutionary theory predicts that strong bottlenecks such as those imposed during the starter culture propagation should purge diversity. However, we currently lack quantitative insights about the composition, functional relevance, and temporal dynamics of strain-level and phage diversity in domesticated microbial communities [12].

Here, we focused on thermophilic starter cultures (RMK202) of a traditional Swiss hard cheese. Thermophilic starter cultures are mainly used with unpasteurized milk and produced at temperatures above 37 °C, which means that other bacteria dominate these communities relative to mesophilic cultures, namely *Streptococcus salivarius* subsp. *thermophilus* (hereafter *S. thermophilus*) and *Lactobacillus delbrueckii* subsp. *lactis* (hereafter *L. delbrueckii*). Genomes of both species have previously been sequenced, and several active phages and CRISPR-Cas defense systems were identified indicating high phage pressure [13].

We hypothesize that the microbial community in this starter culture is shaped by the repeated propagation cycles, and predict that the resulting population bottlenecks, in combination with the homogenous and stable environment (i.e., milk), resulted in a simple microbial community on multiple levels, i.e., species, strain, and phage diversity, as well as genomic content. Here, we used shotgun metagenomics to assess the bacterial and viral diversity that can be maintained across multiple passages of the starter culture RMK202, including historical samples ranging from 1996 to 2019, as well as samples from a propagation experiment, simulating ~60 years of starter culture production. We then tested if the retained diversity contributes to important functional properties by isolating 39 strains representing most of the strain diversity present in the metagenomes and by testing their functional properties in terms of flavor volatile production, bacterial growth, acidification potential, and community resilience. Finally, we extracted all CRISPR spacers and matched them to phages identified across the analyzed samples. Our results show that coexisting strains of the same species are functionally redundant, but differ tremendously in their phage resistance potential. Phages and bacteria seem to stably coexist over time, indicating a complex life cycle potentially relevant for the stable maintenance of the cheese starter culture.

## Methods

### Strain isolation and bacterial counts

Cheese starter cultures (Lot 22.01.2019) were plated on SRY9.3 for *S. thermophilus* [14] and on MR11 (MRS adjusted to pH 5.4 according to ISO7889) for *L. delbrueckii*. Ninety-six colonies per species and per culture were randomly picked and inoculated into liquid media and incubated for 24 h at 37 °C. For genotyping, DNA from 100 μL of culture was extracted using the EtNa DNA isolation method [15] and a mini-satellite PCR was prepared as described in the supplementary methods. Isolates were stored at −40 °C in milk. Colony forming units (CFU/ml) were determined by serial dilution and plating with an Eddy jet spiral plater with counting by a SphereFlash Automatic Colony Counter (both from IUL, Barcelona, Spain) on SPY9.3 and MRS agar.

### Propagation experiment

The samples were propagated to simulate the production of cheese starter cultures. We conducted two passages per week. On Monday, 100 ul of freeze dried sample was inoculated into 10 ml autoclaved biomilk media (BM) and incubated for 18 h at 37 °C. For the second passage on Tuesdays, the pre-culture was inoculated into 10 ml autoclaved BM and incubated for another 18 h at 37 °C. For the final step, 100 ul of the incubated samples were transferred into a freeze dry ampule and stored at −30 °C for at least 1 h. Thereafter, the samples were freeze dried for 7 h until dry. This procedure was repeated six times resulting in 12 propagations for five independent replicates. The number of microbial generations per incubation step were based on CFUs determined at the start and the end of the incubation, ignoring any potential cell lysis. The following formula was used to calculate the number of generations and survival rate in the freeze dry process:

$${{{{{\mathrm{Generations}}}}}} = \frac{{log\left( {\frac{{CFU\left( {t2} \right)}}{{CFU\left( {t1} \right)}}} \right)}}{{log\left( 2 \right)}}$$

$${{{{{\mathrm{survivalrate}}}}}} = \frac{100 * {{{{{\mathrm{CFUbeforefreezedrying}}}}}}}{{{{{{\mathrm{CFUafterfreezedrying}}}}}}}$$

### pH measurements

For the pH measurements we used the hydroplate system (PreSens, Germany). The pH was normalized with pH standards pH 4 and 7. The measurements were done in four replicates for 30 h at 37 °C.

### Historical starter culture data

The acidification and lactate values were gathered from the Agroscope (Liebefeld, Bern) starter culture production archive. Lactate values were irregularly (approximately every 1–2 months) measured by an enzymatic assay previously described [16].

### Genome and metagenome sequencing

Eleven samples of the cheese starter culture RMK202, including historic freeze dried ampules, present working stocks, cheese starter cultures and propagation experiment samples were prepared for shotgun metagenome sequencing. We also prepared 22 and 17 isolates of *S. thermophilus* and *L. delbrueckii* for genome sequencing, respectively. The DNA was isolated as previously explained [17], and Nextera flex libraries prepared and subjected to HiSeq4000 150PE (Illumina) sequencing at the Genomic Technologies Facility in Lausanne, Switzerland and with a rapid barcoding kit on a minION (Nanopore) at the IFIK in Bern, Switzerland.

### Raw read analysis and reference genome analysis

The raw reads were trimmed with trimgalore [18]. The reads were mapped with bwa mem [19]. For the SNV-calling, freebayes-parallel was used [20]. The genomes and metagenomes were assembled with SPAdes [21] and Flye [22]. The Flye assemblies were polished with four rounds of Racon [23] polishing and four rounds of freebayes polishing [20]. Freebayes is an efficient SNV and INDEL caller, which is commonly used to remove all remaing SNV and homopolymer errors from long read genome assemblies by forcing haploid calling (-p 1) and only including the major alleles (-F 0.5). The ANI values were calculated with fastANI [24]. The circular plots were created with circos [25]. In addition, Panaroo [26] and SNPeffect [27] were used to identify core synonymous/non-synonymous mutations. The completeness of the metagenome assemblies was checked with Quast [28], mOTU2 [29] and Busco [30]. pN/pS ratio was calculated with POGENOM [31]. The assemblies were submitted to NCBI and all genes and pseudogenes were annotated with PGAP [32]. The core genome nucleotide phylogenies were constructed as previously described [33].

### SNV calling and strain quantification

For the single nucleotide analysis (SNV), freebayes-parallel was used with the *pooled-continuous* option for metagenomes and the following parameters: “*-C 5 –pooled-continuous –min-alternate-fraction 0.05 –min-coverage 10”*. In addition, to filter the SNVs we used vcftools [34] with the following parameters: “*–minQ 30 –remove-indels –recode –recode-INFO-all*”. Further, only non-synonymous and SNVs in single-copy-genes were considered. The strains relative abundance was quantified by using the mean alternative allele frequency of the SNVs detected solely in one lineage (for details see script).

### GC-MS metabolomics analysis

The metabolic analysis of single *S. thermophilus* and *L. delbrueckii* isolates, pairwise co-cultures, multi-strain co-cultures, and the original starter culture was done after 24 h of incubation in BM at 37 °C. Samples were extracted as previously described by dynamic headspace vacuum transfer in trap extraction (DHS-VTT) [35] and analyzed by gas-chromatography mass spectrometry (GC-MS). For details see supplementary methods. Biolog PM1 plates were applied and measured on the Omnilog according to the producers protocol.

### Phage analysis

All metagenomic reads not mapping against the bacterial metagenome-assembled-genomes (MAGs) were assembled with metaSPAdes, merged, and demultiplexed with cd-hit [36]. The contig coverage was estimated with bedtools [37] and the contigs were manually curated with bandage [38] to obtain full viral genomes (Supplementary Fig. 1). The viral genomes were annotated with Virsorter [39], Phaster [40] and blastn [41]. The phage network was assessed with Vcontact2 [42]. We included all previously described *Streptococcus* phages [43]. The location and fraction of integrated phages was assessed by identifying metagenomic paired-end reads mapping to both phages and bacterial genomes by filtering based on the primary and mate pair mapping location (see script). The fraction was calculated by taking the coverage of these reads in comparison to the mean coverage of the entire contig.

### CRISPR analysis

The CRISPR arrays were annotated with PilerCR [44] and the cas genes with CRISPRcasfinder [45]. The annotation plot was done with genoplotR [46] and the CRISPR repeat identity with the Weblogo online server [47]. Further the metagenomic spacers were extracted with a bash script (see script) and the raw spacers were demultiplexed and rarefied with DADA2 [48].

CRISPR spacer turnover rate calculations were calculated by dividing the number of new CRISPR spacers in propagation experiment samples by the number of generations in that sample (~123 generations). Shared CRISPR spacers are calculated by clustering all extracted CRISPR spacers at 90% identity with cd-hit-est. Metagenomic CRISPR spacers are extracted from the metagenome by using cutadapt [18] to fish out repeat-flanking CRISPR spacers. The protospacer/spacer mapping was done by creating a complete spacer fasta database of repeat-spacer-repeat sequences. The metagenomic raw reads were mapped to this database with bwa mem [19] and the protospacer abundance was quantified by taking the reads that mapped only to the spacer sequence. The spacer abundance was quantified by taking the reads that mapped to the complete repeat-spacer-repeat sequence.

### Analysis and code

All analyses, statistics and plotting was done in R (R Core Team, 2020) and ggplot2 [49]. The complete code is available at: (https://github.com/Freevini/RMK202_analysis). The two MAGs and all *S. thermophilus* and *L. delbrueckii* genomes were deposited under the NCBI Bioprojects PRJNA589532, PRJNA589608 and PRJNA659704. All data used in the analysis is available on Zenodo (10.5072/zenodo.715348).

## Results

### Ongoing genome decay and putative species interactions based on metagenome-assembled-genomes

**Fig. 1:**
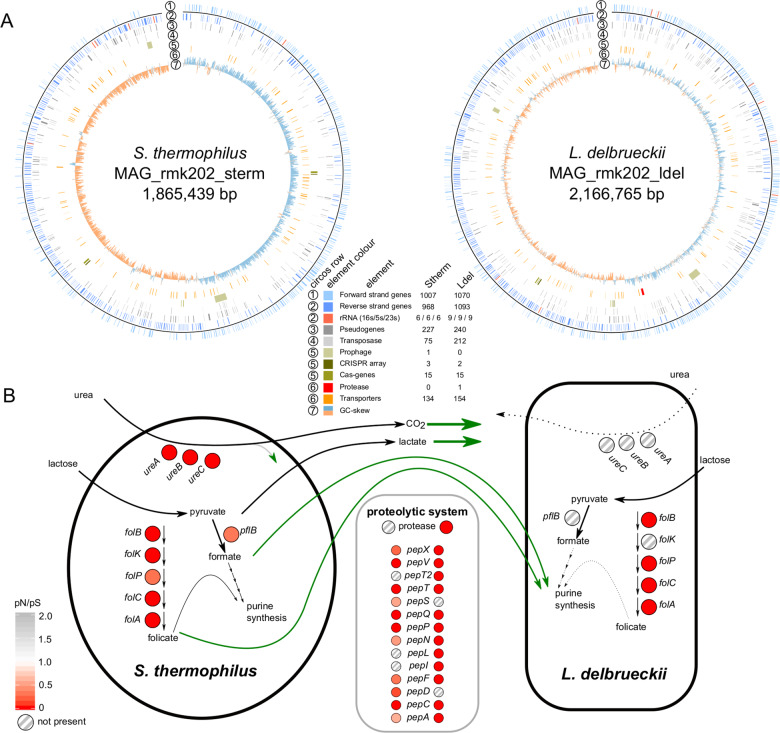
Assembly, annotation, and functional properties of the two metagenome-assembled genomes (MAGs) from the Swiss hard cheese starter culture RMK202. **A** The Metagenome-assembled-genomes of S. *thermophilus* and *L. delbrueckii* with different genetic features highlighted (see legend). **B** Functional properties potentially involved in the metabolic interaction of the two species. The coloring of the circles indicate the ratio of nonsynonymous vs synonymous substitution rates (pN/pS) of the genes, while striped circles indicate the absence.

To obtain a reference for the genomic diversity present in the Swiss hard cheese starter culture RMK202, we applied long read and short read sequencing to the starter culture from 2019. From this combined metagenomic data, we assembled two circular and finished bacterial MAGs (Fig. 1A, Supplementary Fig. 2) according to the MIMAG standards [50]. These MAGs were most similar to *Streptococcus thermophilus* STH_CIRM_19 (ANI = 99.5%, cov = 94.1%) and *Lactobacillus delbrueckii* subsp. *lactis* KCTC 3034 (ANI: identity = 99.1%, cov = 82%), isolated from a French hard cheese Gruyère de Comté in 1963 and from sour milk in 1999, respectively. The two MAGs (hereafter referred to as Stherm_MAG and Lacto_MAG) are 1.9 Mb and 2.2 Mb in length, contain 1975 and 2163 genes, and six and nine complete copies of the rRNA operons, respectively. They both harbored a high number of pseudogenes (Stherm_MAG = 227, Lacto_MAG = 240) and many transposases (*S. thermophilus* = 75, *L. delbrueckii* = 212), which is in line with our hypothesis and previous reports suggesting ongoing genome decay as a result of microbial domestication in dairy communities [51, 52].

We identified multiple CRISPR arrays in both MAGs (Stherm_MAG = 3, Lacto_MAG = 2) indicating previous phage encounters. In addition, we were able to assemble three small and non-conjugative plasmids of which one had a very high and the other two a very low plasmid copy number (PCN) (Supplementary Fig. 3). The three plasmids shared similarity to *Streptococcus suis* plasmid pISU2812 (id = 95%, ~14 kb, 0.18× PCN), and *L. delbrueckii* plasmids pWS58 (id = 95%, 10 kb, 3.2× PCN) and pJBL2 (id = 90%, 8 kb, 0.09× PCN), but contained few genes with annotations (Supplementary Fig. 3).

In yogurt fermentation, *S. thermophilus* and *L. delbrueckii* subsp. *bulgaricus* have been described to engage in a mutualistic interaction by provisioning nutrients to each other [53, 54]. Similar metabolic interactions may also occur between the two corresponding species in cheese starter cultures (Fig. 1B). Genes necessary for folate, lactate, and formate production were all present and under purifying selection in Stherm_MAG (pN/pS < 1, Supplementary Fig. 4). In Lacto_MAG, these pathways were either incomplete or completely absent (Fig. 1B). In turn, *L. delbrueckii* encoded the only casein-cleaving protease in the system (Fig. 1B). Proteases are assumed to be of crucial importance for peptide cleavage and flavor development in cheese production [55]. Together, these results suggest that both species are maintained due to metabolic complementation and potentially positive effects on each other.

### Temporal stability of a two-species community in Swiss hard cheese starter cultures

**Fig. 2:**
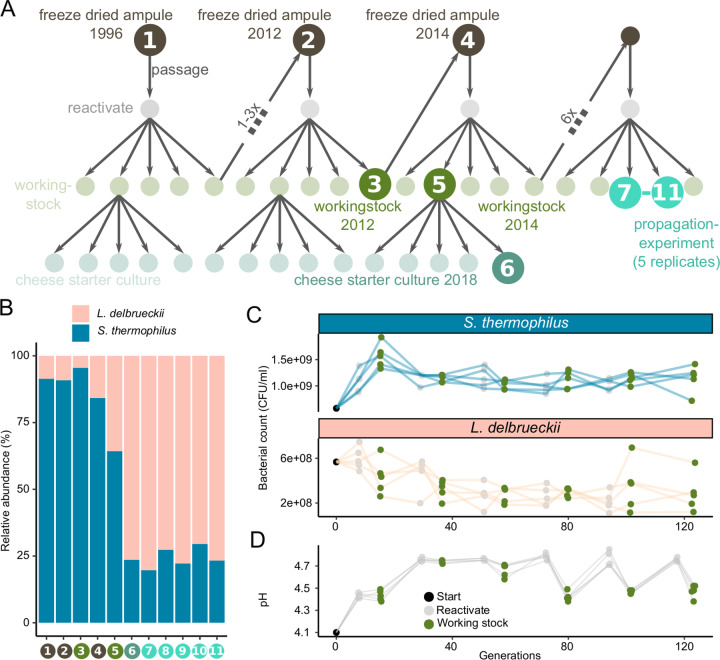
Metagenomic sampling design and species abundance. **A** The starter culture propagation scheme as applied in the cheese starter culture production. The samples subjected to metagenomic sequencing are indicated by darker colors and labelled with numbers. Every propagation cycle includes a freeze drying (lyophilization), reactivation, and working stock step. From the working stock, commercial starter cultures for weekly shipments to cheesemakers are produced. The propagation experiment was carried out in the same way as in the production plant and in five replicates corresponding to samples 7–11. The numbers between the working stock (x) indicate the number of cycles in between. Between 1996 and 2012 there are between 1 and 3 propagtion cycles. **B** The relative abundance of the two bacterial species in the 11 starter cultures samples (as illustrated in Fig. 2A). **C** Bacterial counts throughout the propagation experiment for both species and the five replicates (lines are colored according to species and points according to samples within Fig. 2A). **D** Acidification potential throughout the propagation experiment, as measured by pH reached after 18 h incubation at 37 °C in milk.

To assess the effect of continuous passaging on the starter culture’s community structure and population dynamics, we documented the temporal dynamics of the community members. Cheese starter cultures are maintained by a systematic propagation scheme in order to minimize compositional shifts (Fig. 2A, Supplementary Fig. 5). In addition to the already characterized starter culture from 2019, we analyzed six historical samples from 1996 to 2018 (9–15 propagation steps). Further, we continued to propagate the starter culture 12 times in five replicates, which is equivalent to 60 years of starter culture maintenance, or ~123 bacterial generations, considering a survival rate of 71.5% after freeze-drying (Fig. 2A, Supplementary Fig. 6). All samples were short read sequenced to a depth of 5–18 million reads. We did not identify any additional community members, apart from the two species characterized in Fig. 1.

To estimate the relative abundance of the two species, we mapped the metagenomic reads against the two MAGs (Fig. 2B). The historical samples revealed more variation in relative abundance of the two community members than the five replicates of the propagation experiment, most likely due to inconsistent incubation time (varied historically between 18 and 24 h) rather than real community differences. Colony counts of both *S. thermophilus* (mean = 1*10^9^, SD = 2*10^8^) and *L. delbrueckii* (mean = 3*10^8^, SD = 1*10^8^) remained similar throughout the 12 propagations (Fig. 2C), and all samples stably acidified to an average pH of 4.6 ± 0.2, (Fig. 2D and Supplementary Fig. 6). The stable metabolic activity of both community members is further supported by the consistently high acidification rates (Supplementary Fig. 7) and constant ratios of D-lactate (i.e., *Streptococcus*) to L-lactate (i.e., *Lactococcus*) (mean = 30% ± 10%; Supplementary Fig. 8) in the culture production plant since 1996. Note, that the metagenomic relative abundance underestimated the *L. delbrueckii* abundance in comparison to the CFU count. This is most likely due to incomplete cell lysis of *S. thermophilus*. The abundance of three identified plasmids also remained similar throughout the sampling period (Supplementary Fig. 9). Together, these findings suggest that the two species stably coexist in the hard cheese starter cultures with little variation in abundance or phenotypic properties across time.

### Low levels of intra-species diversity with a few stably coexisting *S. thermophilus* strains

While the starter culture community consists of only two stably coexisting species, there might be further diversity at the strain-level. To assess the extent of intra-species diversity in the analyzed cheese starter cultures, we quantified single nucleotide variants (SNVs) across the core genes of the two community members. The fraction of variable sites differed between the two species, but showed little variation across the 11 metagnomes. For *S. thermophilus*, we found an average of 0.4% ± 0.002% (i.e., 4136 SNVs) variable sites and for *L. delbrueckii*, we found an average of 0.0005 ± 0.0002% (i.e., 130 core genome SNVs) variable sites (Supplementary Fig. 10). This is much lower than what has been reported for natural bacterial communities such as the human gut microbiome (~4%) [56] or the bee gut microbiome (~10%) [33] and seems to confirm that the repeated propagation in a stable and homogenous environment and the existence of strong population bottlenecks during freezing-propagation cycles result in low levels of strain-level diversity.

**Fig. 3:**
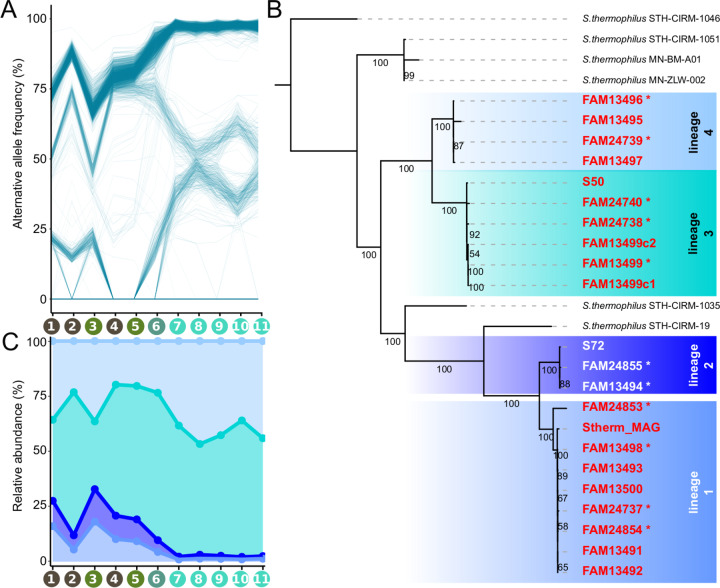
Strain-level diversity of *S. thermophilus* in cheese starter cultures. **A** Alternative allele frequencies of all *S. thermophilus* SNVs over the metagenomic samples. Recurring SNVs from different samples are connected with a line. Clustering of lines indicates a large amount of SNVs with similar frequencies suggesting genomic coupling. Sample labels on the *x*-axis correspond to samples highlighted in Fig. 2A. **B** The phylogeny of the isolated *S. thermophilus* strains based on maximum likelihood analysis on 1788 core genes. The isolates split into four lineages indicated by different color shadings. Strains sequenced with Nanopore are labelled with an asterisk. Values on branches indicate bootstrap values (100 replicates). **C** The relative abundance of each of the four sub-lineages of *S. thermophilus* across the 11 metagenomes as based on the average frequency of lineage-specific SNVs identified on the basis of the isolates in Fig. 3B.

To identify coexisting strains of *S. thermophilus*, we looked at SNV frequencies across the 11 metagenomic samples. SNVs with similar frequencies in several independent samples are likely to be physically linked on the same genomic element and consequently contained in the same strains [57]. We found that the SNVs clustered into three (Supplementary Fig. 11) discrete phases (i.e., putative strains, Fig. 3A), They were consistently present across the 11 metagenomic samples, except for one phase which transiently disappeared in two consecutive samples (Fig. 3A, sample 4 and 5), indicating that it fell below the detection limit (<0.05 alternative allele frequency). This analysis was not conducted for *L. delbrueckii*, given the low number of SNVs in this species.

To further characterize the variation within each of the two species, we cultured bacterial strains from the 2018 starter culture (sample 6). We genotyped the isolates using a mini-satellite region and sequenced their genomes using short-and long-read sequencing. This resulted in 22 and 17 high quality and finished genomes of *S. thermophilus* and *L. delbrueckii*, respectively (Supplementary Table 1). Overall, the assembled genomes explained 97% core genome SNVs detected across the 11 metagenomes. The unexplained SNVs did not belong to any of the identified phases and were of lower abundance (Supplementary Fig. 12), indicating that we have cultured nearly the complete microbial diversity of the analyzed cheese starter cultures.

The isolated *L. delbrueckii* strains were highly similar (ANI > 99.86) to each other, containing 1732 core genes with 130 SNVs identical to the 130 SNVs identified across the 11 metagenomes (Supplementary Fig. 13). Th accessory genome contained only 118 genes, most of which had no annotation (*n* = 53) or encoded transposases (*n* = 59), which are generally very common in Lactobacillus genomes (mean = 163, sd = 56) and are hard to assemble [58].

In contrast, the genomes of the *S. thermophilus* isolates were less similar to each other. The core genome consisted of 1788 genes with 4116 SNVs. Phylogenetic analysis revealed that the *S. thermophilus* isolates clustered into four well-supported lineages, which were not monophyletic but separated by two French hard cheese (Comté) isolates (Fig. 3B). *S. thermophilus* harbored slightly more accessory genes (354) which were mostly annotated, similar as in *L. delbrueckii*, as hypothetical proteins (41%) or transposases (24%). A few accessory genes (*n* = 21, i.e., 6%), however, encoded functions related to amino acid transport and polysaccharide biosynthesis (Supplementary Fig. 14), including eight glycosyl- and sugar transferase genes involved in cell wall modifications of *S. thermophilus* and known for their role in phage sensitivity [59, 60]. The genomes from different lineages were largely syntenic (Supplementary Fig. 15). This explains, together with the relatively high similarity among the strains, why the MAG represented a single circular and non-chimeric genome (Fig. 1A).

Based on the isolated strains, we determined SNVs specific to each of the four lineages, and used their average frequency to estimate the abundance of each lineage across the metagenomic samples (Fig. 3C). This revealed that all four lineages stably coexist in the cheese starter cultures, but that lineage 3 and 4 were predominant in particular in the propagation experiment.

### Interspecies interactions and phenotypic redundancy among strains of the cheese starter cultures

While the composition of the community is simple, the two species and the multiple coexisting strains may contribute important cheese-related functional properties (i.e., rapid acidification and flavor volatiles production) and express different ecological traits that facilitate their stable coexistence (i.e., growth or metabolic potential). We experimentally assessed the functional differences across species and strains by looking at three important phenotypic properties, namely the growth rate, acidification rate, and the flavor volatiles production. We not only tested each isolate separately, but also included different pairwise strain combinations, a mix of all strains, and the original starter cultures.

**Fig. 4:**
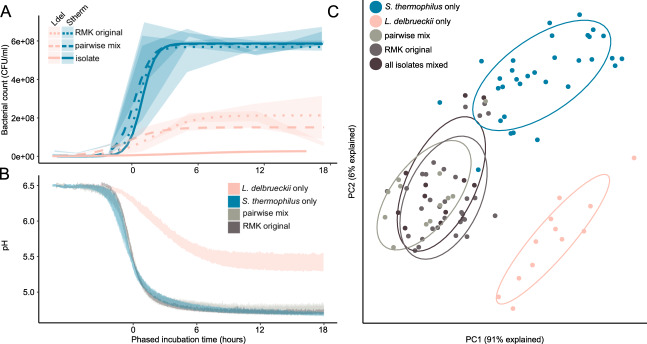
Phenotypic properties of individual strains, pairwise combination of strains, and original starter culture. **A** Colony forming units (CFUs) of *S. thermophilus* and *L. delbrueckii* over 18 h of growth when cultured alone, in pairwise combinations, or in the original starter cultures (RMK). The ribbons illustrate the interquartile range and the lines the modeled growth curves. **B** Acidification curves of the same samples. The ribbons illustrate the min and max pH of the different samples. **C** Principal component analysis of the metabolic profiles after 24 h of growth at 37 °C. Different treatments are highlighted in colors and with the surrounding eclipse.

For both species, all tested strains showed highly similar growth and acidification rates in mono-cultures. *S. thermophilus* isolates grew and acidified rapidly reaching a carrying capacity of 5.9*10^8^ CFU/ml, and decreasing the pH to 4.7 already 3 h after the lag phase (Fig. 4A, B). In contrast, *L. delbrueckii* isolates grew and acidified significantly slower reaching a carrying capacity of 2.8*10^7^ CFU/ml, and decreasing the pH to 5.5 only 7 h after the lag phase (Fig. 4A, B). Notably, culturing the two species together resulted in a tenfold increase in the amount of *L. delbrueckii* from 2.8*10^7^ CFU/ml to 2.2*10^8^ CFU/ml, in all strain combinations as well as in the original starter culture, suggesting a strong positive effect of *S. thermophilus* on *L. delbrueckii* growth. In contrast, neither were the cell counts of *S. thermophilus* higher in any of the co-culture conditions in comparison to the axenic cultures, nor did we observe a difference in acidification.

To identify potential differences in the formation of flavor volatiles among the individual strains and between different co-culture combinations, we compared the untargeted volatile profiles measured by dynamic headspace trap extraction on the GC-MS (DHS-VTT) as previously optimized for the measurement of cheese flavor volatiles [35]. The measurements were conducted after 24 h of growth in milk. Principal component analysis identified three distinct clusters, two encompassing all strains of each species when grown alone, and one representing all co-cultures (Fig. 4C). This suggests that strains of the same species produce similar metabolic profiles, and that the metabolic profile changes when the two species are grown together. However, the number and combination of strains did not affect the metabolic profile of the co-culture. Biolog plate experiments revealed no differences in the utilization of milk related carbon sources and all strains had the same gene complement for amino acid biosynthesis corroborating that strains are metabolically identically to each other (Supplementary Fig. 16).

Taken together, these results suggest that interbacterial interactions between the two species modulate the flavor volatile profiles of starter cultures, but that any combination of strains reconstitutes the acidification properties and volatile profile of the starter cultures.

These findings reject our hypothesis that strain-level diversity contributes to flavor volatile diversity and suggest that the strains are functionally redundant in terms of important cheese making properties as well as ecological traits.

### Highly variable phage resistance mechanisms in isolated genomes

**Fig. 5:**
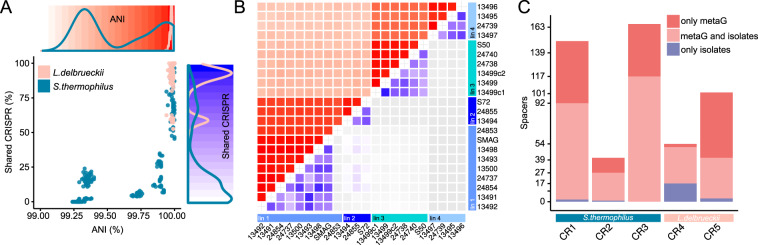
CRISPR spacer diversity of *L. delbrueckii* and *S*. *thermophilus*. **A** The correlation of fraction of shared CRISPR spacers and ANI of all *L. delbrueckii* and *S. thermophilus* with the corresponding densities and heatmaps on the *x*-and *y*-axis. **B** The heatmap of the genomic and CRISPR spacer diversities of *S*. *thermophilus* illustrated with ANI (top heatmap; from white to red) and percent shared CRISPR spacers (bottom heatmap; from white to blue). **C** The amount of metagenomic (metaG) and genomic (isolates) CRISPR spacers according to the five arrays.

Ecological theory predicts [61] that functional redundancy without niche specialization prevents stable coexistence, which is in contrast with our results. We hypothesize that phage predation may contribute to the maintenance of low levels of strain diversity in the analyzed cheese starter culture, similar as suggested for mesophilic cheese starter cultures [11]. We can infer such interactions from genomic data, as bacteria store a memory of past phage encounters in their genome by integrating short spacer sequences into CRISPR arrays [62]. We found three previously described CRISPR-regions (CR) in the analyzed *Streptococcus* genomes, namely CR1 (type II-A), CR2 (type III-A) and CR3 (type II-A) [63, 64] and two in *Lactobacillus*, CR4 (type IC) and CR5 (type III-A) [65] (Supplementary Fig. 17). Despite little genetic divergence and accessory gene content variation, we found high variability in the number and identity of the CRISPR spacers in both species. A total of 236 and 92 unique CRISPR spacers were identified for *S. thermophilus* and *L. delbrueckii*, respectively. Each strain carried between 23 and 83 spacers (Supplementary Fig. 18), 75 spacers were only present in a single strain (Supplementary Fig. 19), and none of the spacers was shared among all *S. thermophilus* strains (Supplementary Fig. 20). Interestingly, the fraction of shared spacers between two genomes rapidly decreased with increasing genomic distance (Fig. 5A). Moreover, *S. thermophilus* strains of the same lineage shared between 58 and 76% of their spacers, which is surprisingly little considering that the genomes within a lineage were nearly identical based on ANI (ANI = 99.999%, Fig. 5B). *S. thermophilus* CRISPR spacers present in only one strain tended to lie closer to the leader (Supplementary Fig. 21), where new spacers are usually acquired [66].

To get a better understanding of the temporal dynamics of CRISPR spacer turnover, we extracted all reads containing CRISPR spacer sequences from the metagenomes, and assigned them to one of the five CRISPR arrays of *Lactobacillus* and *Streptococcus*, based on their conserved repeat sequence motifs (Supplementary Fig. 17). We identified a total of 354 and 131 dereplicated metagenomic CRISPR spacers for *S. thermophilus and L. delbrueckii*, respectively. Despite culturing the large majority of strain-level diversity (97%), 121 (34%) and 64 (49%) spacers were not present in any of the isolated genomes of *S. thermophilus and L. delbrueckii* (Fig. 5C). Moreover, 17 *S. thermophilus* and 38 *L. delbrueckii* spacers were only found in the metagenomes of the propagation experiment. From these newly acquired spacers, and the known bacterial generation time, we estimated a mean CRISPR spacer turnover rate of 0.028 (SD = 0.020) and 0.086 (SD = 0.047) spacers per bacterial generation for *S. thermophilus* and *L. delbrueckii*, respectively. This is much lower than what has been observed in previous experiments where *S. thermophilus* was challenged with pac phages and acquired novel matching spacers in the corresponding CR1 and CR3 arrays [67]. Together, these results suggest a continuous turnover of CRISPR spacers and ongoing bacteria-phage interactions.

### Two abundant and persistent *Streptococcus* phages in the cheese starter cultures

**Fig. 6:**
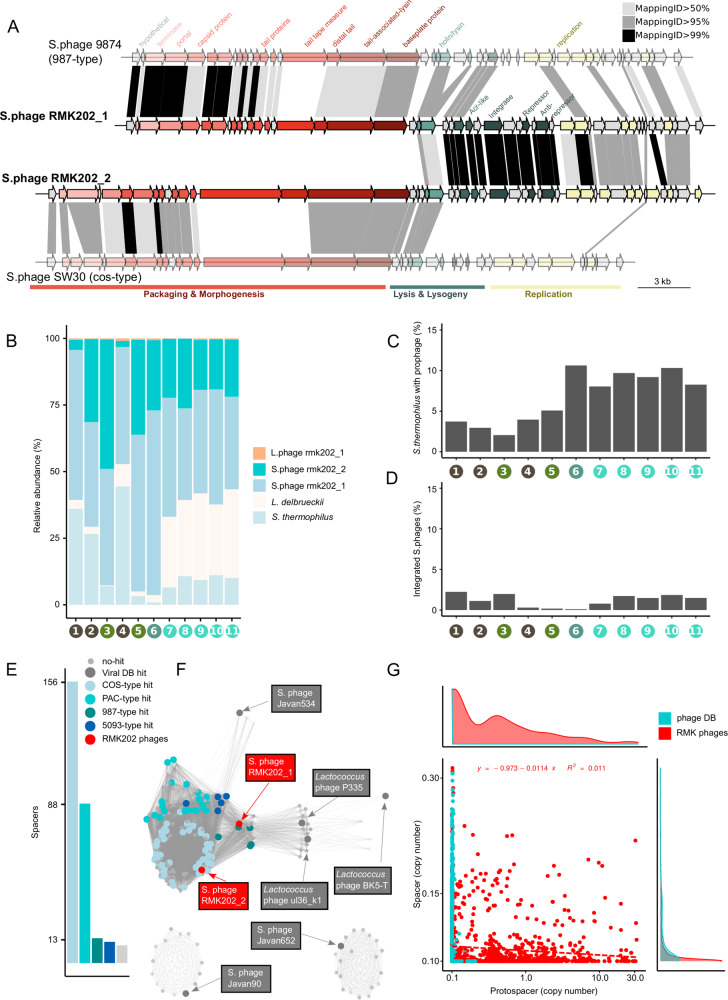
Characteristics of the phages identified in the cheese starter cultures. **A** Gene annotation of the two *Streptococcus* starter culture phages, RMK202_1 and RMK202_2, and the two closest relatives (illustrated in lighter colors). Protein similarity between genes are indicated in gray (80–95% identity) and black (95–100%). **B** Relative abundance of bacteria and phages over all metagenomic samples based on genome read coverage. **C** Fraction of *Streptococcus* genomes with an integrated phage as based on the read coverage of phage-bacteria spanning regions relative to the coverage of the *S. thermophilus* genome. **D** Fraction of *Streptococcus* phages which show signs of integration as based on the read coverage of phage-bacteria spanning regions relative to the coverage of the *Streptococcus* phage genomes. **E** The number of spacers mapping against the different phage types. **F** The *Streptococcus* phage network with the protospacer containing phages colored or labeled according to phage type. **G** The spacer abundance vs the protospacer abundance from all phage spacers. The database specific linear regression and distributions are indicated in the figure and the axis figures accordingly.

Our CRISPR spacer analysis suggests high rates of CRISPR spacer turnover and continuously high phage pressure. In order to characterize the viral community in the cheese starter cultures, we assembled all metagenomic reads that did not map against the bacterial MAGs (2–29%, Supplementary Fig. 22). A total of 23 non-redundant contigs were assembled on the basis of which we could reconstruct three complete phage genomes (rmk202_1–3, Supplementary Fig. 1). Adding these phages to our bacterial reference genomes (MAGs and plasmids, see above) resulted in the mapping of the large majority of all metagenomic reads (99.97%, SD = 0.02%, Supplementary Fig. 23). Comparison with the viral RefSeq database indicated that phages rmk202_1 and rmk202_2 clustered with cos-type and 987-type phages of *Streptococcus* (Fig. 6A, Supplementary Fig. 24), respectively, whereas phage rmk202_3 clustered with the *Lactobacillus* phage JCL1032 (Supplementary Fig. 25).

Metagenomic read recruitment revealed that both *Streptococcus* phages were highly abundant, markedly surpassing bacterial numbers in several of the analyzed cheese starter cultures (1–110 phage copies per bacteria; Fig. 6B, Supplementary Fig. 26). In contrast, the *Lactobacillus* phage was much less abundant with an average of 0.1 phage copies per bacteria (SD = 0.1×). Both *Streptococcus* phages showed little genetic variation: we only detected six mutations across the 11 samples despite the fact that both phages were sequenced very deeply (Supplementary Fig. 27). Remarkably, despite being highly divergent in most of the genome, the two *Streptococcus* phages shared a high degree (>99% nucleotide identity) of similarity in the lysis and lysogeny gene cluster coding for holin, lysin, integrase, repressor, and anti-repressor proteins suggesting a recent recombination event and a similar temperate lifestyle (Fig. 6A, Supplementary Table 2) [68–70]. The phage rmk202_2 was found to be integrated in the genome of only one of the 22 characterized *S. thermophilus* strains (FAM24739, Supplementary Table 1). Moreover, we identified metagenomic read pairs that mapped with one mate to the phage and the other to the *S. thermophilus* genome, providing further evidence for genomic integration. Based on the location of the mapped phage-bacteria spanning read pairs, we were able to identify four alternative integration sites (Supplementary Fig. 28). Moreover, a subset of the read pairs unambiguously mapped to each of the four lineages indicating that all lineages serve as host for the *Streptococcus* phages (Supplementary Fig. 29). Based on the relative number of such phage-bacteria spanning read pairs, we estimated that between 2 and 11% of all *S. thermophilus* cells carry an integrated phage (Fig. 6C), but that only between 0.1 and 2% of all *Streptococcus* phage genome copies are integrated (Fig. 6D). This highlights that the phages are active in the starter cultures and likely produce large numbers of phage particles.

Of the total 354 metagenomic spacers, 35% matched to one of the two *Streptococcus* phages, 46% matched to viral RefSeq phages, and 3% targeted bacteria (including prophages, plasmids). Only 16% of all metagenomic spacers could not be assigned to any known sequence. Each *Streptococcus* isolate, including the one containing the integrated phage, contained 3–15 spacers against the two assembled *Streptococcus* phages (Supplementary Fig. 30). The presence of multiple spacers targeting the same phage suggests repeated encounters of that phage, as the integration of additional spacers is facilitated during CRISPR-mediated phage interference (known as interference-driven spacer acquisition [71]). The matching spacers were located throughout the CRISPR arrays (Supplementary Fig. 31), suggesting both relatively old as well as more recent acquisition events. Moreover, the majority of newly acquired *S. thermophilus* spacers (15 of 17) in propagation experiment match to the two phages. Based on the above estimated spacer turnover rate (0.024 spacers/generation), and taking into account the distance of the spacer from the leader, we estimated that the first spacers targeting the two phages were acquired between 818 and 1412 generations ago (Supplementary Fig. 32), corresponding to more than 117 passages, which is long before the extraction of the undefined starter culture in the 70 s (~27 passages). This illustrates long-lasting interactions between these two phages and their bacterial hosts.

Bacterial metagenomes are biased against phage particles due to the DNA extraction method. Therefore, we cannot exclude that additional lytic phages were present in the analyzed cheese starter cultures. In line with this, a large fraction of the identified spacers matched sequences present in the viral RefSeq database (46%) including phages from all four major *Streptococcus* phage types (Fig. 6E/F). These spacers were distributed across the CRISPR arrays suggesting that the bacteria in the starter cultures have been experiencing continuous and ongoing phage pressure (Supplementary Fig. 31).

Surprisingly, we did not detect a clear negative correlation between spacer and protospacer abundance (Fig. 6G). Under the assumption that spacers represent resistant bacteria and protospacers active phages, this result does not support that bacteria-phage interactions in the starter cultures are governed by kill-the-winner dynamics [72]. This is in contrast to the previous finding in mesophilic starter cultures used to make Gouda cheese [11]. The exceedingly high numbers of phage copies suggest an ongoing proliferation of the phage most likely from a temperate source. Given the high abundance of the two phages in the starter cultures, despite the prevalence of matching CRISPR spacers, we assume that the phages can evade the CRISPR defense system [60, 73, 74]. We know that the type III CRISPR-Cas system can tolerate silent prophages [75]. Moreover, both phage genomes encode anti-CRISPR (Acr) proteins AcrIIA3 [76] and AcrIIA6 [77] known to inhibit type II CRISPR-Cas systems after mutualstic co-infection (Fig. 6A). Also the annotated tracrRNA of the type II-A CRISPR-Cas system has recently been shown to mitigate autoimmunity but at the cost of reduced bacteriophage immunity [78]. Alternatively, the two phages may follow a carrier state or chronic infection lifestyle, in both cases the CRISPR defensive system would not be effective, as the phage particles persist either absorbed to the bacterial cell envelope (carrier state) [79] or within the bacterial cytoplasm (chronic infection) [80, 81].

In summary, our results show that two phages are consistently present in the analyzed starter culture and outnumber bacterial cell counts indicating active phage replication without evident impact on the stability or phenotypic properties of the community.

## Discussion

The presented results show that the analyzed Swiss hard cheese starter culture is dominated by a simple and highly stable bacterial community composed of *S. thermophilus* and *L. delbrueckii* subsp. *lactis*. Genomics, metabolomics, and growth experiments suggest that the two species engage in similar metabolic interactions as found for *S. thermophilus* and *L. bulgaricus* in yogurt fermentation [54]. Of note, a positive growth effect was only observed for S*. thermophilus* on *L. delbrueckii*, but not vice versa. To determine if the two species have reciprocal benefits on each other and hence can truly be considered as a cooperative association, a more detailed analysis of their growth in co-culture will be needed.

Despite the simple composition at the species level, strain-level diversity may contribute important functional properties to undefined cheese starter cultures such as flavor volatile diversity or community resilience to phage invasion or environmental disturbances [3]. We found relatively little strain-level diversity in the analyzed cheese starter cultures when compared to other microbiomes [33, 82–84]. Three different factors are likely to contribute to this: (1) milk is a comparably simple and homogenous growth environment, (2) cheese starter cultures are closed systems providing little opportunities for microbial migration, and (3) population bottlenecks occur when cultures are propagated and lyophilized. For *L. delbrueckii*, almost no genetic diversity was detected, while for *S. thermophilus* a few genetically distinct variants co-existed across the analyzed samples. Cultured isolates of these strains exhibited highly similar acidification rates, growth characteristics, and volatile profiles suggesting that they are functionally redundant in phenotypic traits relevant for their ecology and the cheese making process.

So, why are strains of the same species still maintained in cheese starter cultures? First, divergent strains may partition the available resources by niche specialization when in co-culture. Second, the population bottlenecks and growth differences among strains may not be sufficient to eliminate low-abundant strains. And third, interactions with phages may facilitate the maintenance of strain-level diversity in the community. Our genomic analysis revealed that in both species most of the genomic variation between strains is found in the CRISPR spacers, the adaptive immune response of bacteria against phages. A large diversity of spacers were found across the isolated strains and the metagenomes. Moreover, new spacers were acquired during the course of our propagation experiment. Based on these results, we conclude that undefined starter cultures must be continuously exposed to phages suggesting that bacteria-phage interactions play an important role in cheese starter cultures.

While our metagenomic preparation was not targeted toward the sequencing of viral particles, we identified two highly abundant *Streptococcus* phages in our dataset. These phages neither showed genetic variation across the analyzed samples (spanning hundreds of bacterial generations) nor did their abundance change much over time. Also, we did not find a negative correlation between protospacer-spacer abundances. Based on these results, we conclude that the bacteria and phages do neither engage in an arms-race [85, 86] nor is their interaction governed by kill-the-winner dynamics [11, 87]. Instead, we observed that some of the phage copies are integrated into the host genome in a subset of the bacterial population, speaking in favor of a piggy-back-the-winner strategy, in which the phages persist in the community by following lysogenic life cycle [88].

Another remarkable feature of the two phages is that they are both maintained at high abundance in the starter cultures (often exceeding the bacterial abundance), without any obvious impact on bacterial growth or the cheese making process and despite the presence of a large number of matching CRISPR spacers in the bacterial population. Together, these findings suggest that the two phages have longstanding associations with their bacterial hosts, can evade the CRISPR-based immunity of the host, and exhibit a lifestyle that may be different from other temperate phages. While phages are generally seen as a potential risk for starter cultures, it is conceivable that domesticated phages such as those found here, could also have beneficial functions for the host. They could provide protection against lytic phages via superinfection exclusion [89], or hinder the establishment of susceptible competitor strains. By entering the lytic cycle at environmentally less favorable time points for the bacteria [90], such as suboptimal pH [91] or low nutrient [92] conditions, the phages may also play a direct role in cheese making as bacterial lysis is believed to enhance the flavor profile of cheese due to the liberation of intracellular proteases [93, 94].

Although our analysis is based on a single cheese starter culture, our work shows that domesticated microbial communities provide fascinating systems to learn about eco-evolutionary dynamics of phage-bacteria interactions. Future studies on the ecology of these phages and their role in the cheese making process could help to design artificial microbial communities in food biotechnology and beyond.

## Supplementary information

SUPPLEMENTARY_FIGURES

SUPPLEMENTARY_TABLE_1

SUPPLEMENTARY_TABLE_2

SUPPLEMENTARY_METHODS
