# Supplementary material for: Functional strain redundancy and persistent phage infection in Swiss hard cheese starter cultures
Source: ISME J. 2021 Aug 6;16(2):388–99. doi: 10.1038/s41396-021-01071-0 (PMC8776748; doi:10.1038/s41396-021-01071-0)
Supplement: Supplementary file 1 — SUPPLEMENTARY_FIGURES [file 41396_2021_1071_MOESM1_ESM.pdf]

Supplement figures: Functional strain redundancy and persistent phage infection in Swiss hard cheese starter cultures

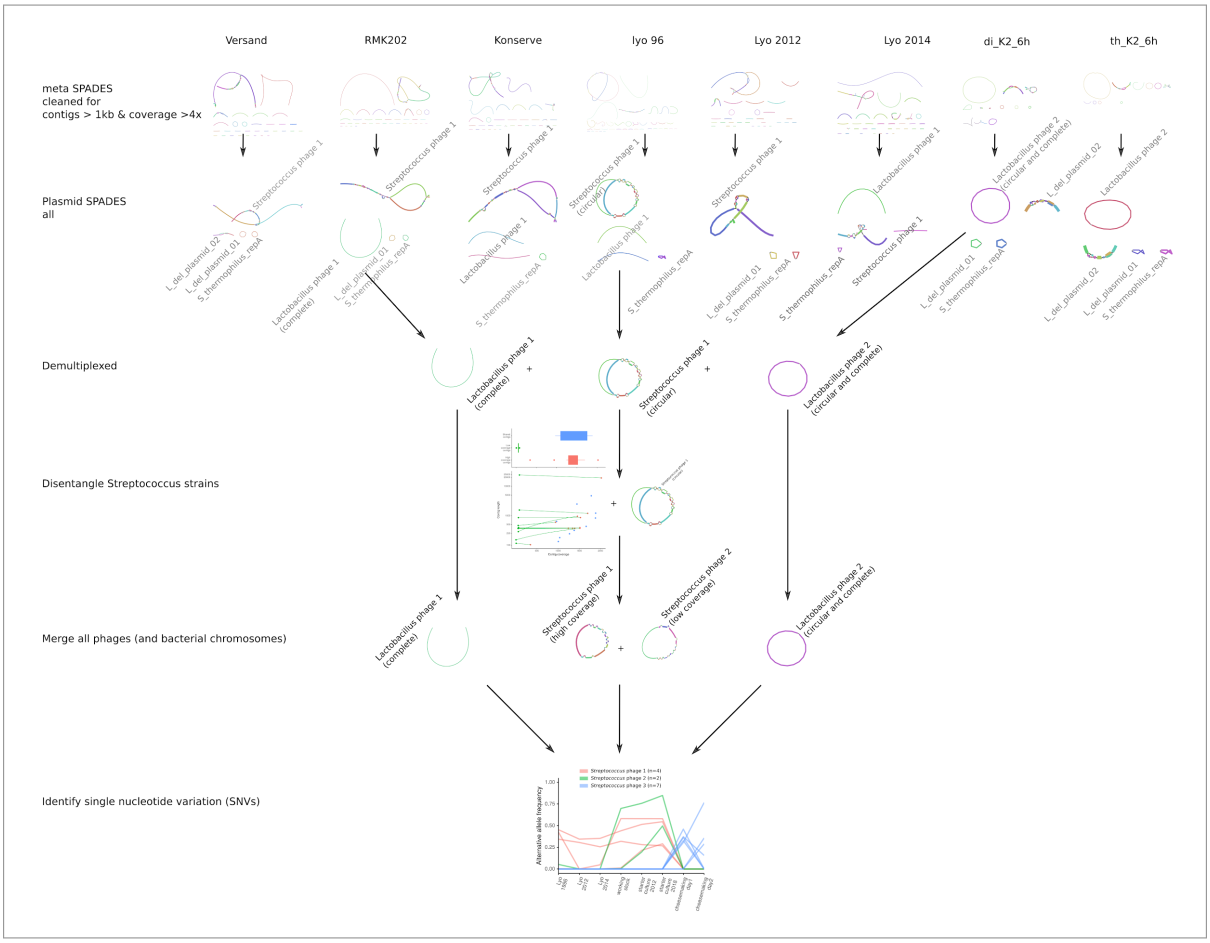

Figure 1. The curated phage assembly with all *involved* steps: 1) metaSPAdes assembly of unmapped reads. 2) compare with plasmidSPAdes assembly of unmapped reads 3) demultiplex all assemblies with cd-hit. 4) Disentangle *Streptococcus* phages based on contig coverage in bandage (Wick et al. 2015). 5) Merge the disentangled phages and check continuous mapping. 6) Identify single nucleotide variations (SNVs) on the curated phage genomes

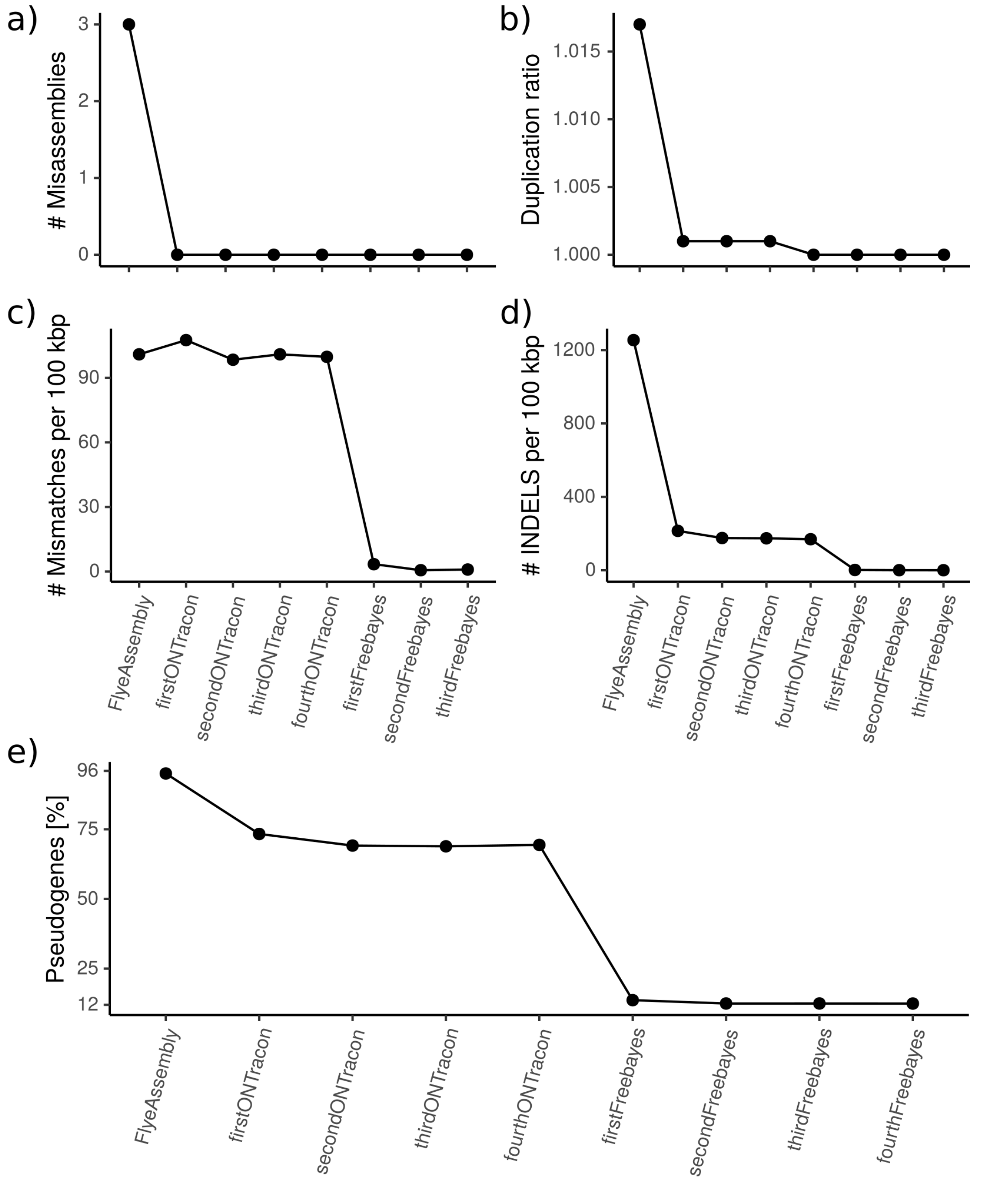

Figure 2. Polishing of the metagenome-assembled-genomes (MAGs). The quality can be illustrated by the steady decrease of a) misassemblies, b) duplication rate, c) mismatches, d) INDELS, and e) pseudogenes over the four Racon-based polishing steps and the four Freebayes-based polishing steps.

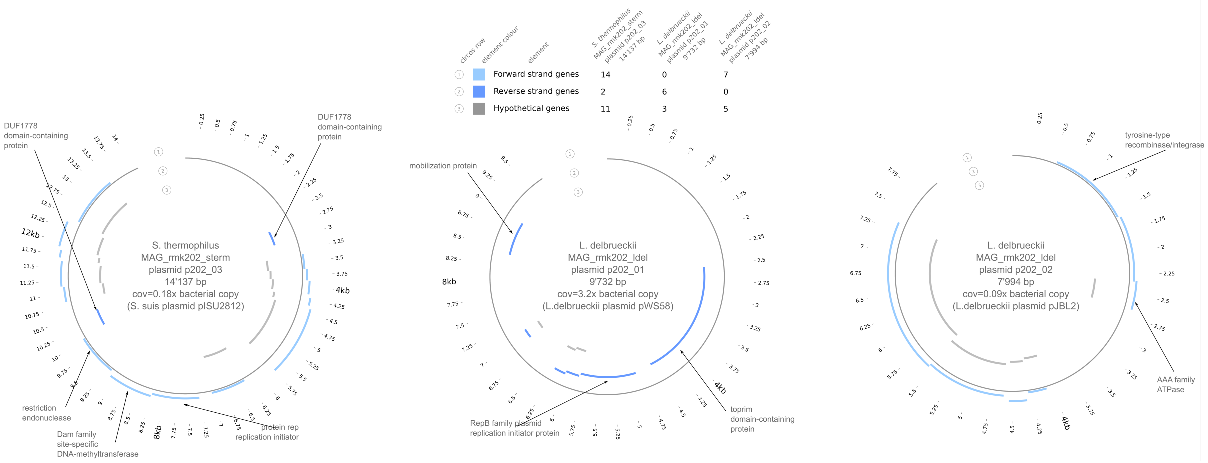

Figure 3. The metagenome-assembled plasmids. The plots include gene annotations, and are labelled with plasmid name, size, coverage (relative to bacterial host), and closest blast hit.

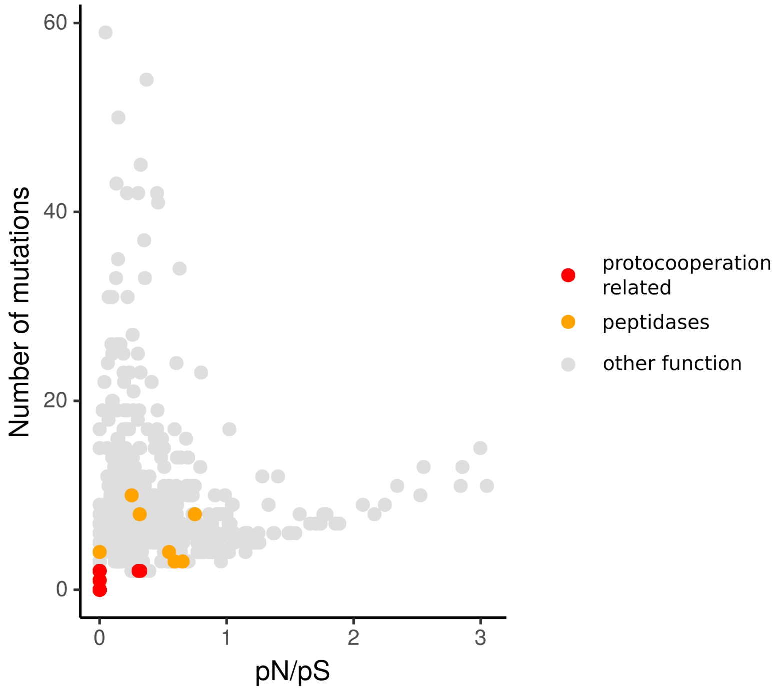

Figure 4. pN/pS ratios and number of mutations of all genes of the *L. delbrueckii* and *S. thermophilus* MAGs. The genes related to protocooperation and the peptidases are colored accordingly.

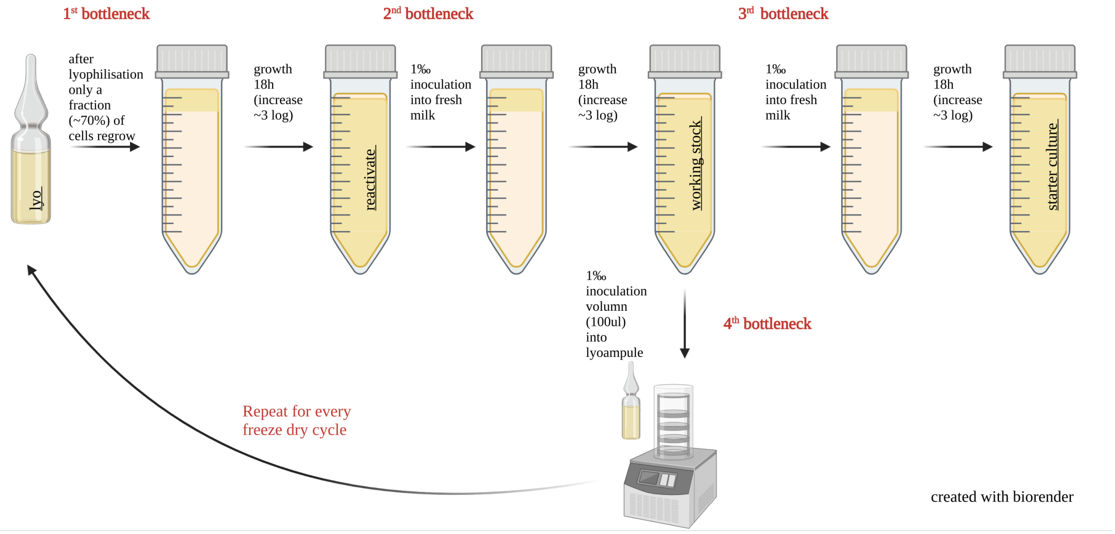

Figure 5. Cheese starter culture propagation diagram with the extent and amount of bottlenecks highlighted in red. This figure was created with biorender.

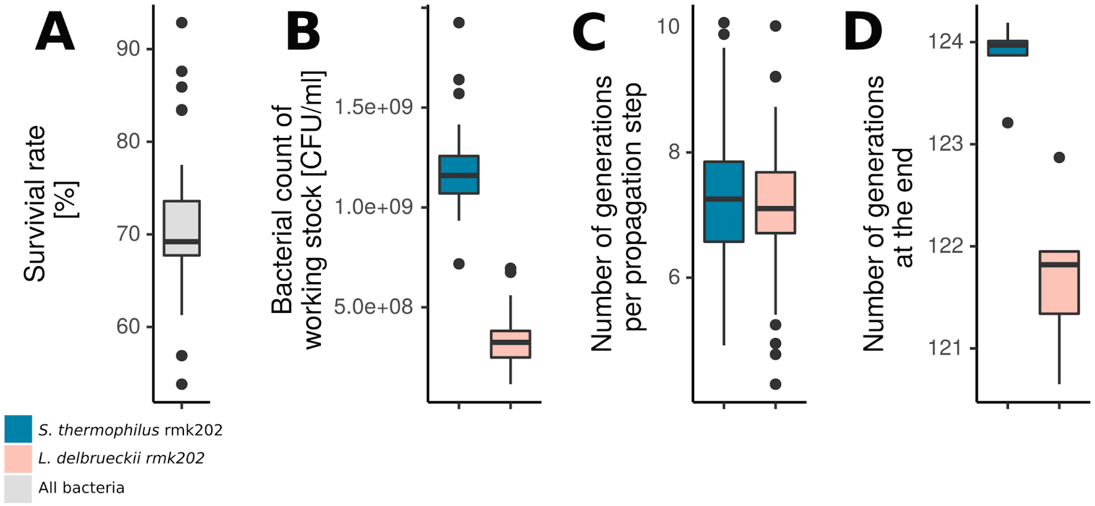

Figure 6. The analysis of the phenotypic data of the propagation experiment. A) The overall survival rate of the bacteria after freeze drying. B) The bacterial counts in CFU/ml of the working stocks for both species. C) The number of generations per passage over the entire experiment for both species. D) The final number of generations at the end of the evolution experiment per species.

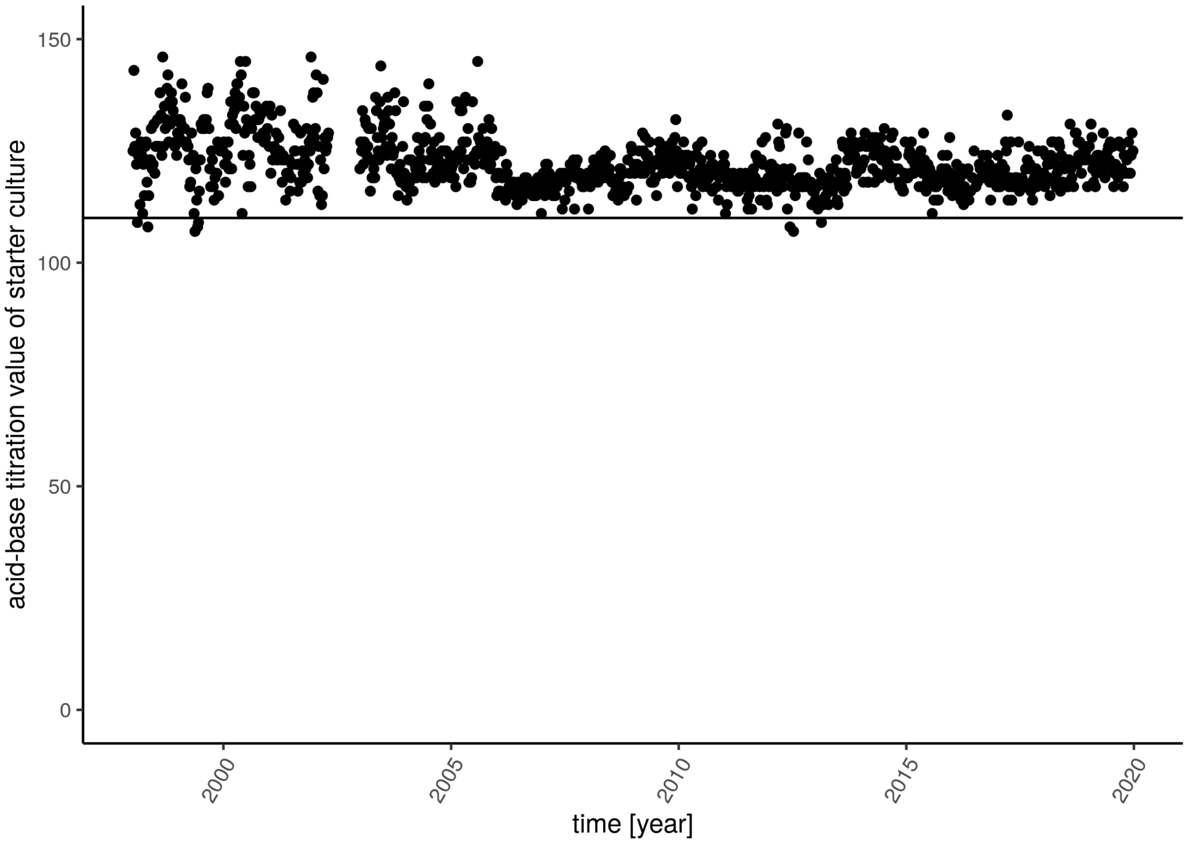

Figure 7. The acid-based titratable value of the starter culture RMK202 ranging back to 1996. The titratable acidity is measured after incubation for 18 h at 37°C in milk. The black line is the lowest minimum accepted value.

Figure 8. The percent of D-lactate to L-lactate measured after 18 h of incubation at 37°C in milk. D-lactate is produced by *L. delbrueckii* and L-lactate by *S. thermophilus*. Measurements were irregularly conducted between 2003 and 2015. Dates are illustrated on x-axis.

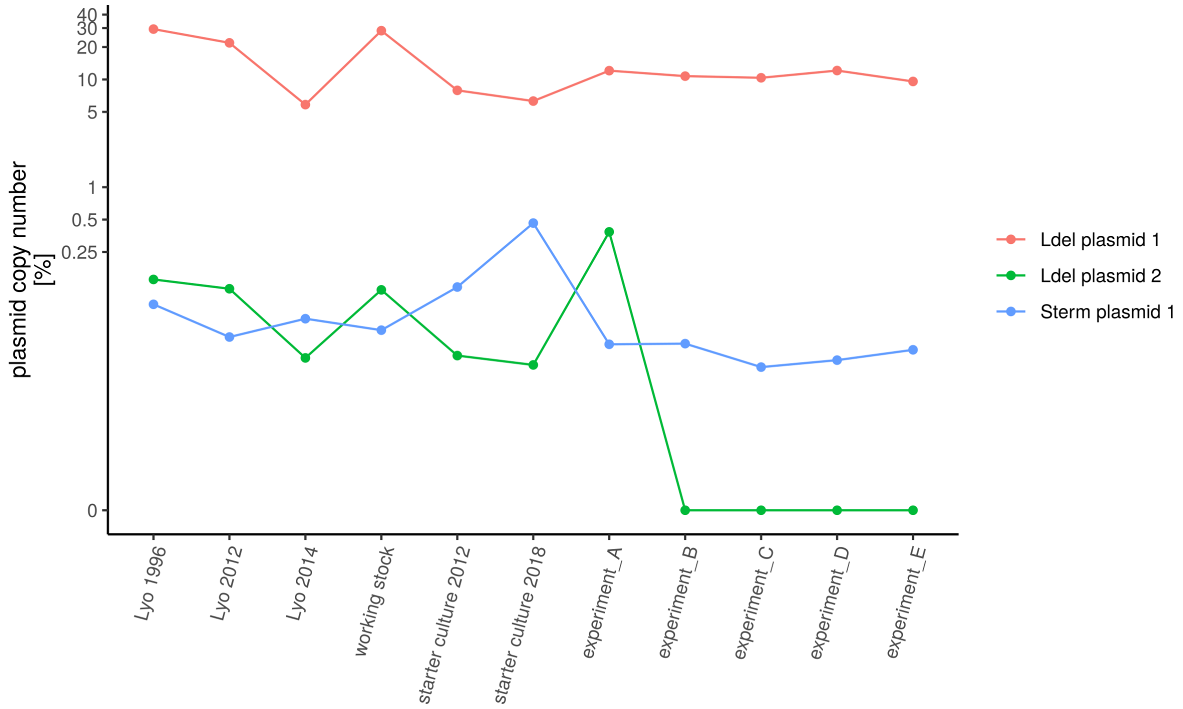

Figure 9. Plasmid copy number of all detected MAG plasmids in all metagenomic samples. The copy number is calculated in relationship to the host bacterial abundance.

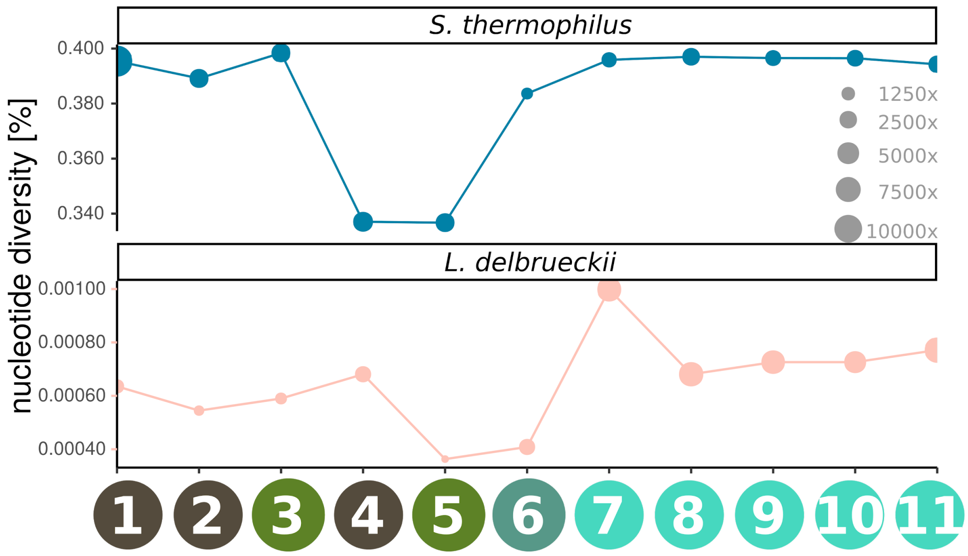

Figure 10. The fraction of variable sites (nucleotide diversity) in the housekeeping genes of the *S.thermophilus* (top) and *L. delbrueckii* (bottom) over the 11 metagenomic samples. The coverage on the individual samples is indicated with the size of the point. (The legend for the samples on the x-axis are illustrated in Fig. 2A).

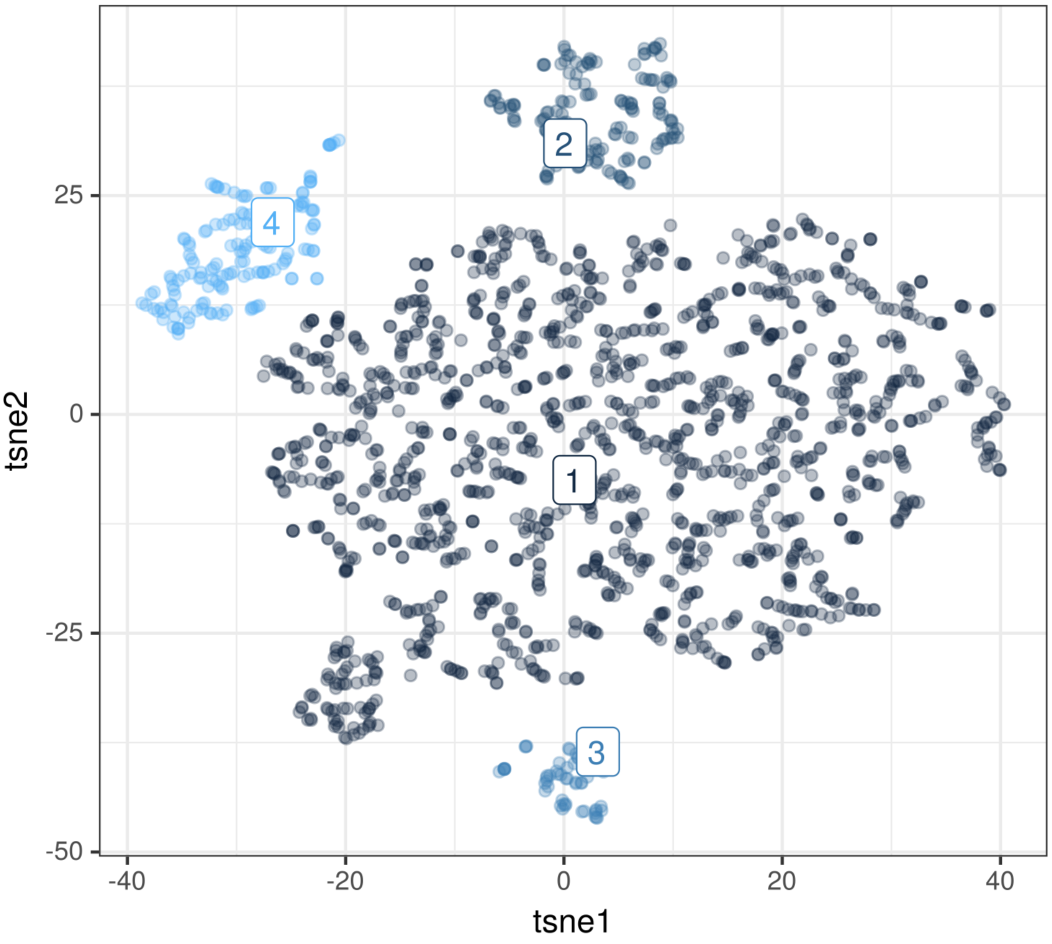

Figure 11. The Tsne clustering of all metagenomic *S. thermophilus* SNVs.

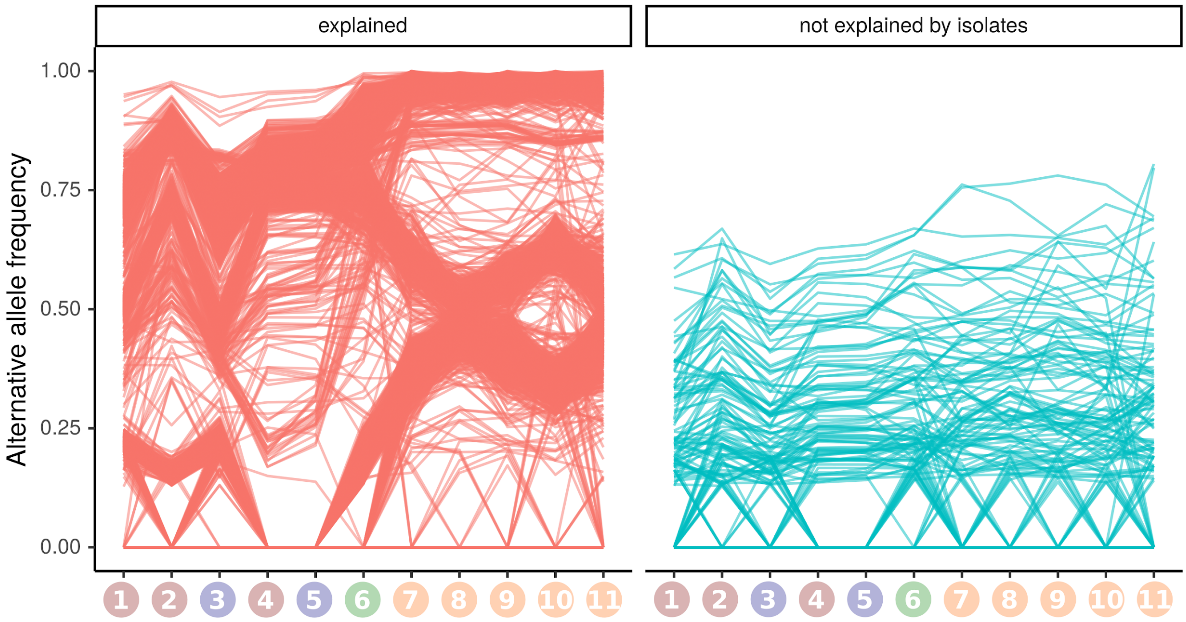

Figure 12. The alternative allele frequency of all *S. thermophilus* SNVs that are explained (97%) and not explained (3%) by the isolates. The x-axis labels correspond to the sample annotations in figure 2A.

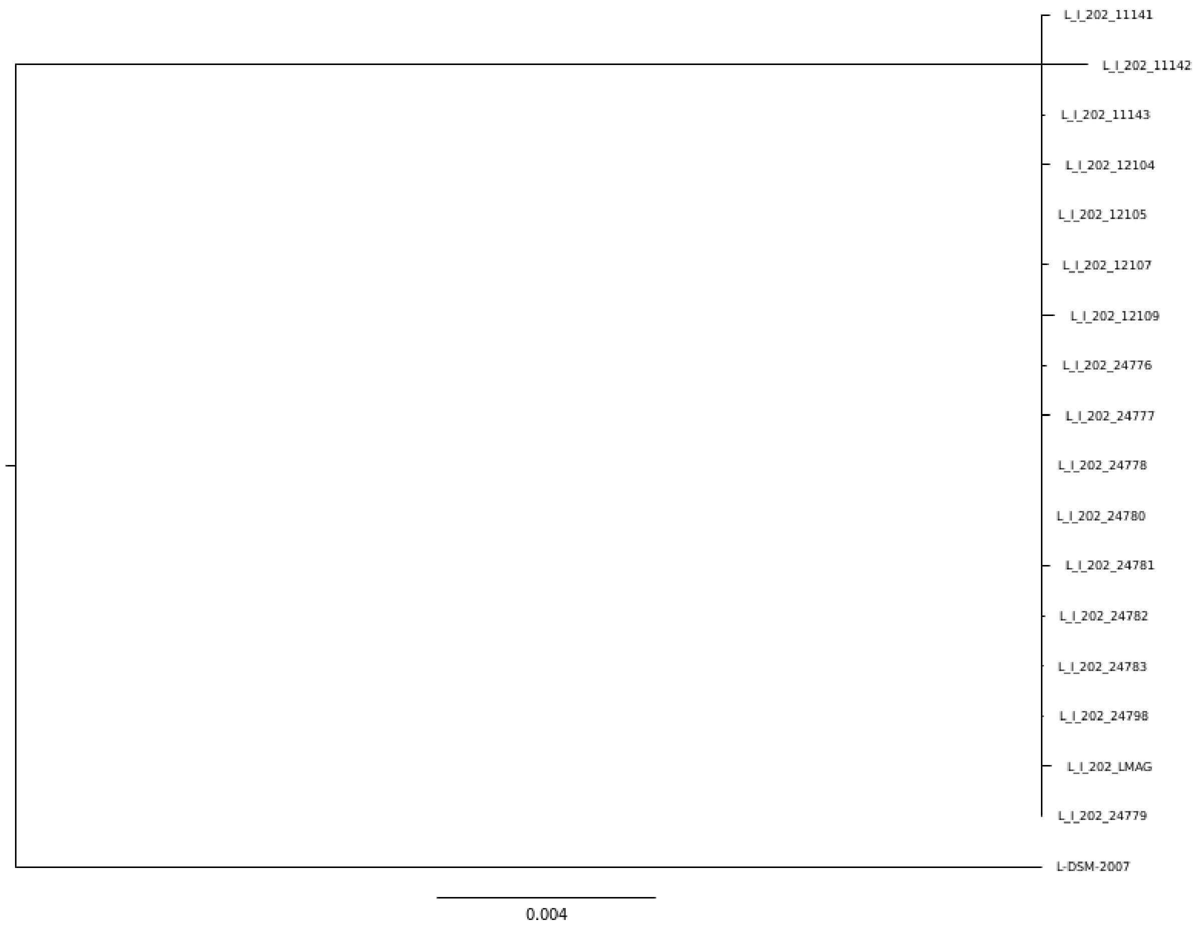

Figure 13. Phylogeny of all RMK202 *L. delbrueckii* isolates with the *L. delbrueckii subsp. lactis* type strain DSM-2007 as outgroup. The phylogeny is based on 1596 core genes.

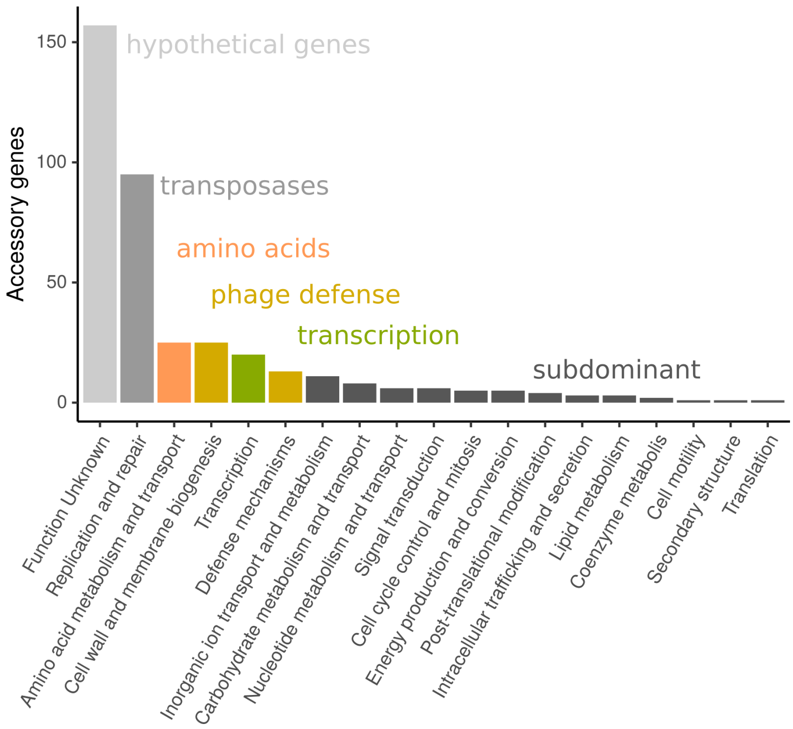

Figure 14. The accessory genes of *S. thermophilus* grouped into different COG categories.

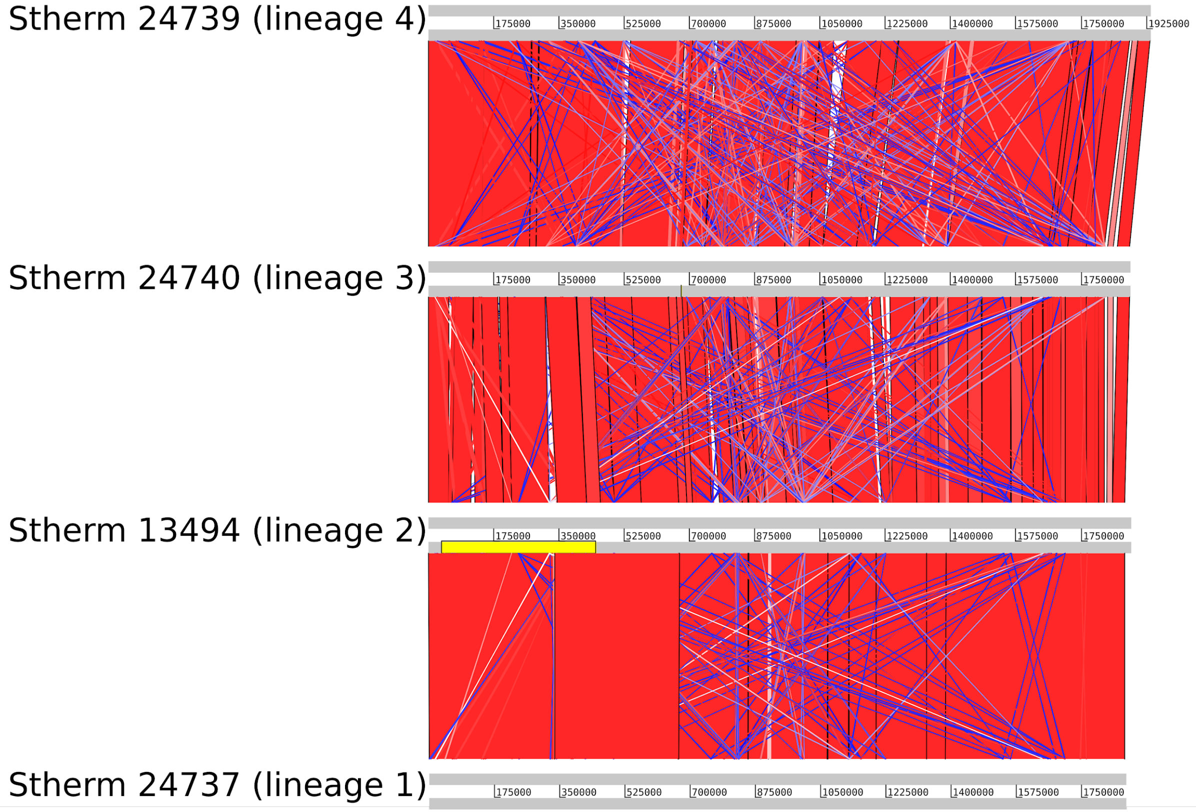

Figure 15. Artemis collinearity plot of the representative genomes from all four lineage of *S. thermophilus*. The regions are selected for min 1000bp and min 90% nucleotide identity. The collinear regions illustrated are colored according to orientation (red=same orientation, blue=reversed).

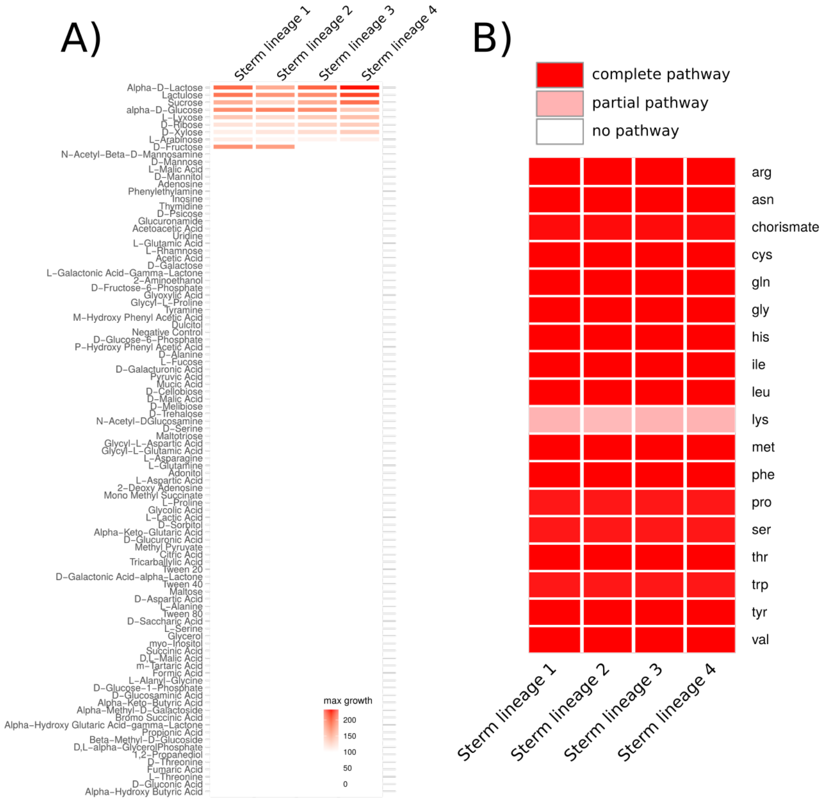

Figure 16: The experimentally and in silico tested resource utilization of the four lineages of *S. thermophilus* . A) The experimental growth on PM1 Biolog plates illustrating the utilization of 95 different carbon sources. Only variation on fructose was observed. However fructose does not occur in milk. B) The in silico predicted amino acid synthesis ability. No variation was observed between the different *S. thermophilus* lineages.

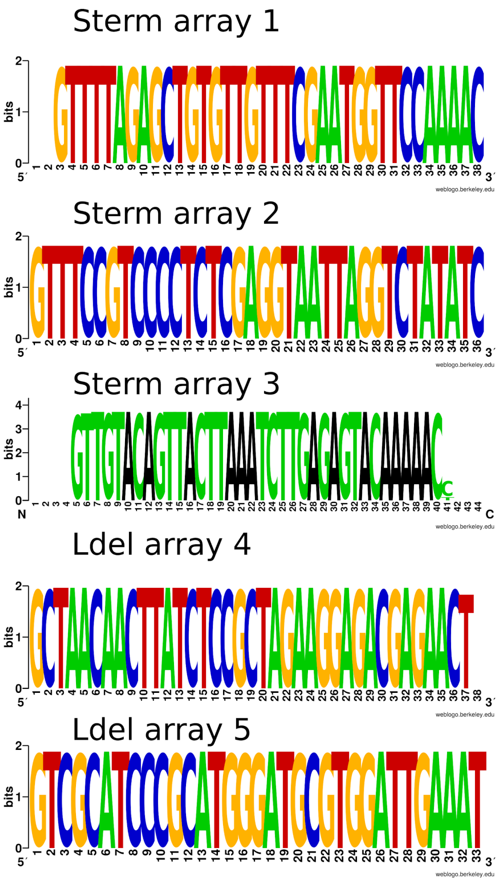

Figure 17. CRISPR repeat conservation over all assembled genomes is illustrated in the weblogo. (Sterm= *S. thermophilus* and Ldel= *L. delbrueckii)*

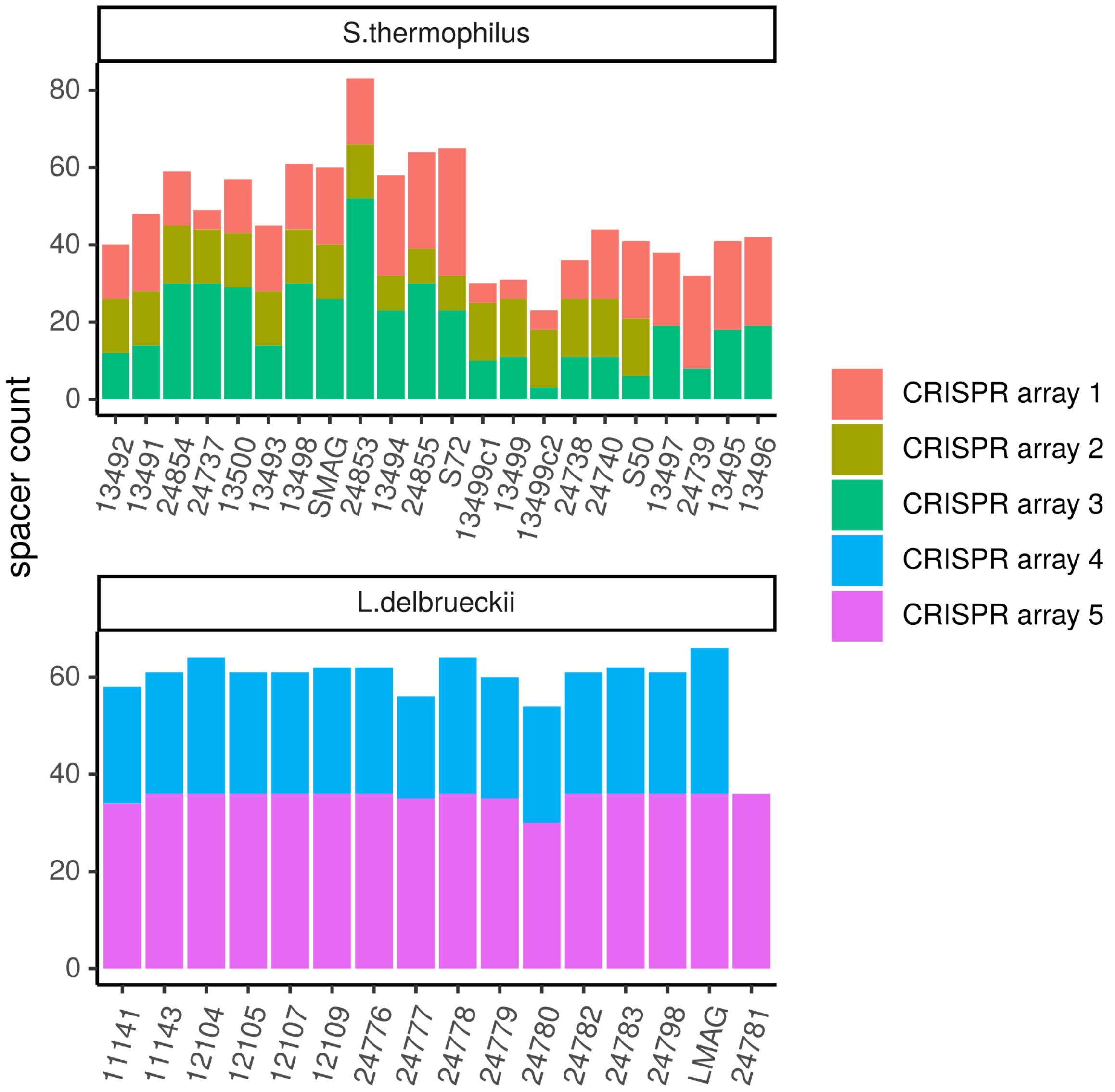

Figure 18. Number of spacers per *S. thermophilus* (top) and *L. delbrueckii* (top) strains. Colors indicate the respective CRISPR array. (*L. delbrueckii* 24781 does not contain any CRISPR array 4)

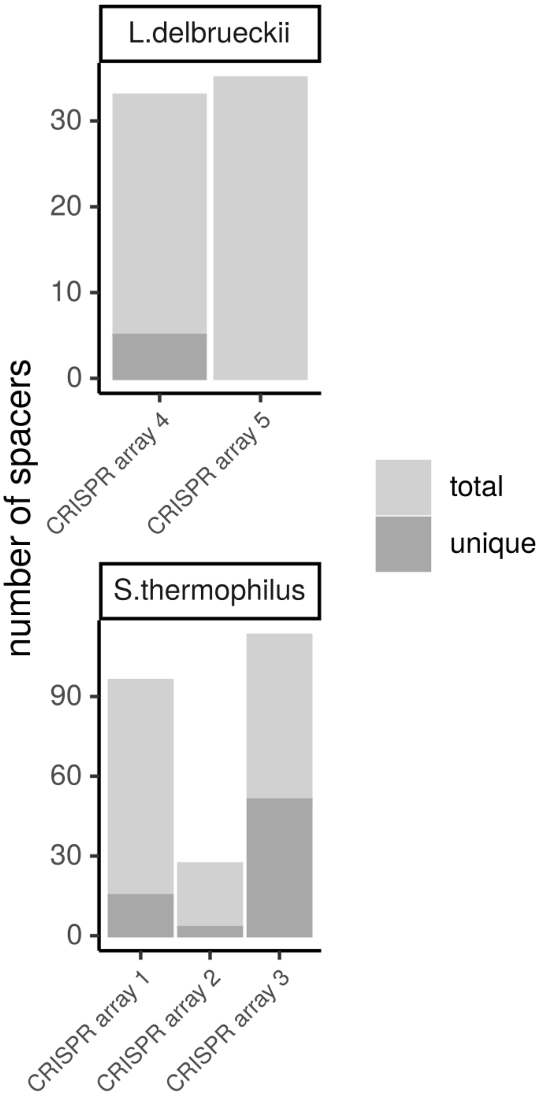

Figure 19. The overall number of spacers in the five arrays. The fraction of unique spacers are indicated in darker gray.

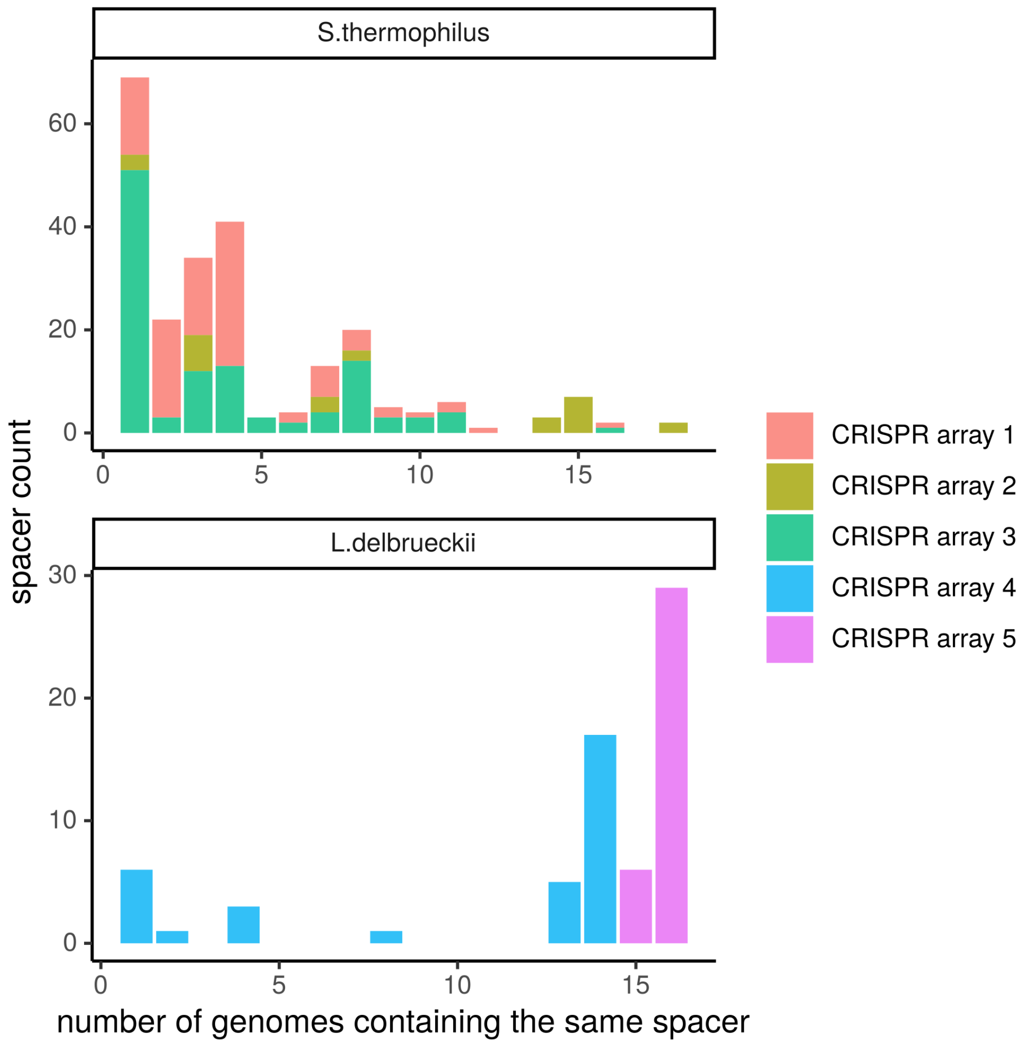

Figure 20. Number of genomes containing the same spacer. The colors indicate the CRISPR array the spacer is associated with.

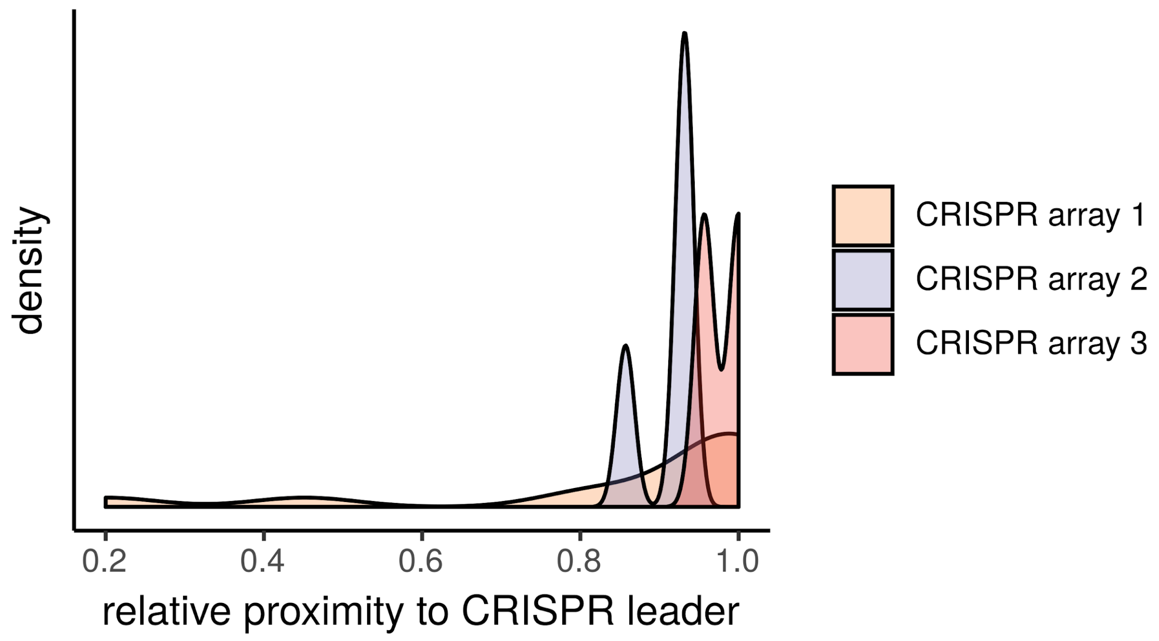

Figure 21. The relative proximity to the CRISPR leader of all unique CRISPR spacers isolated from the *S. thermophilus* genomes.

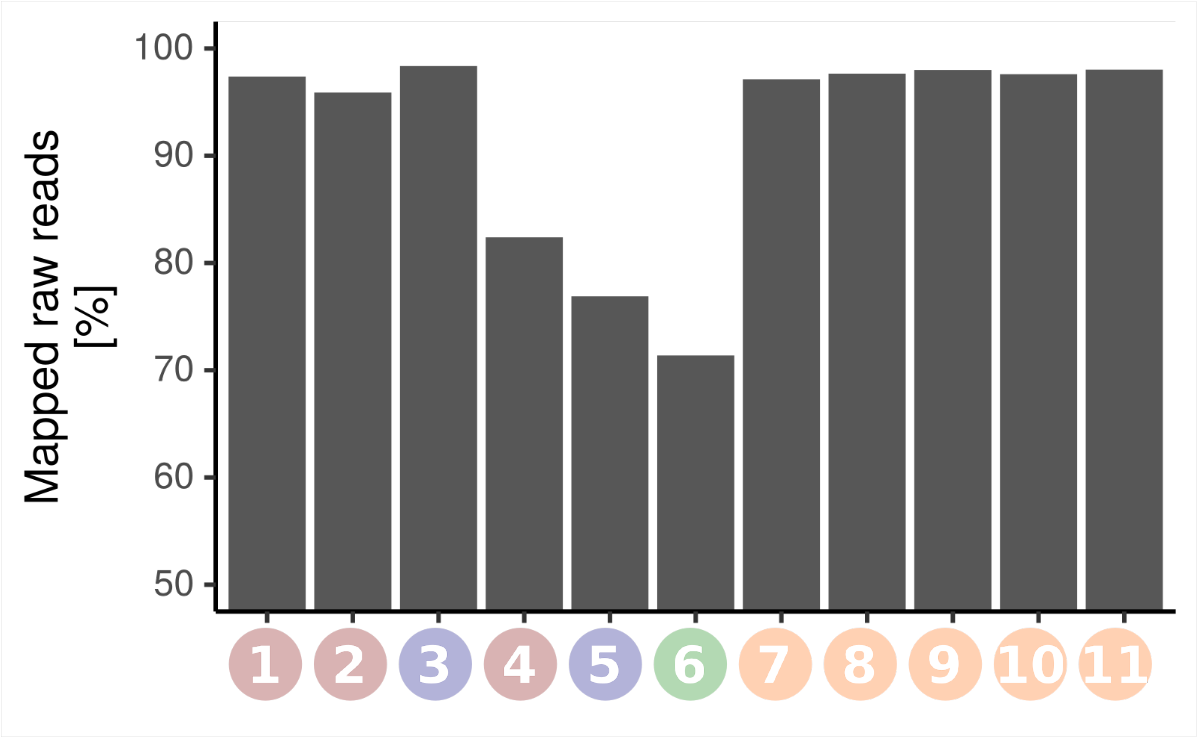

Figure 22. Percent of mapped raw reads for the different metagenomic samples against the MAGs. The sample labels on x-axis are chronologically and described in more detail in Fig. 2A.

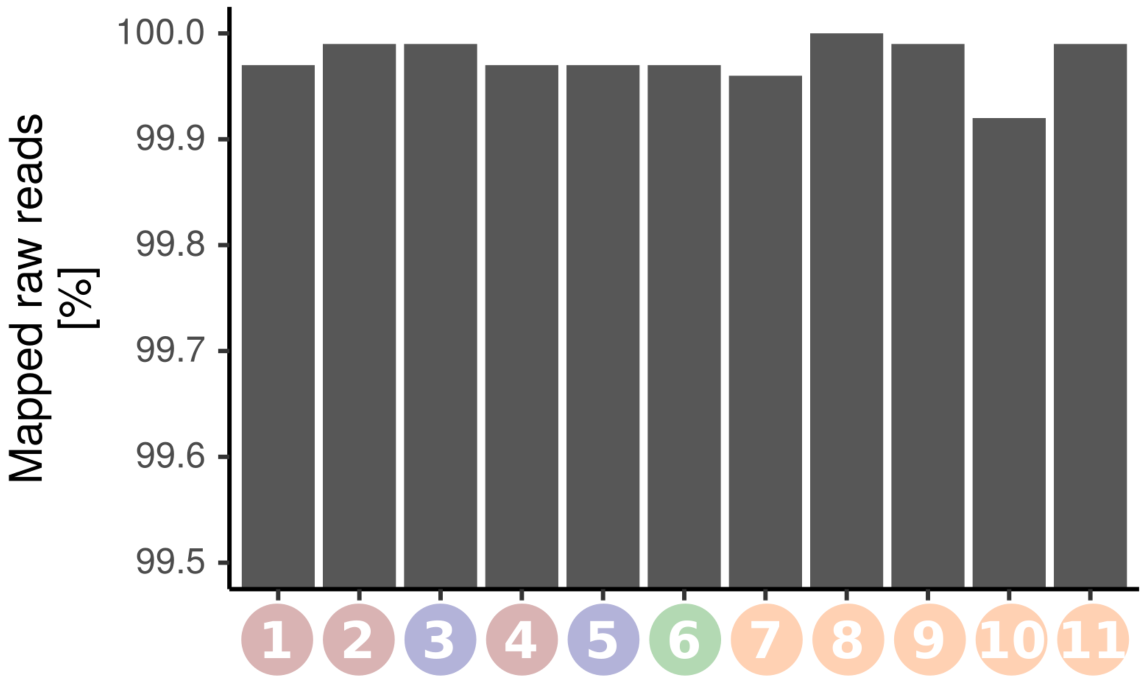

Figure 23. Percent of mapped raw reads for the different metagenomic samples (mean=99.97%, sd=0.02%). Sample labels on x-axis are chronologically and described in more detail in Fig. 2A.

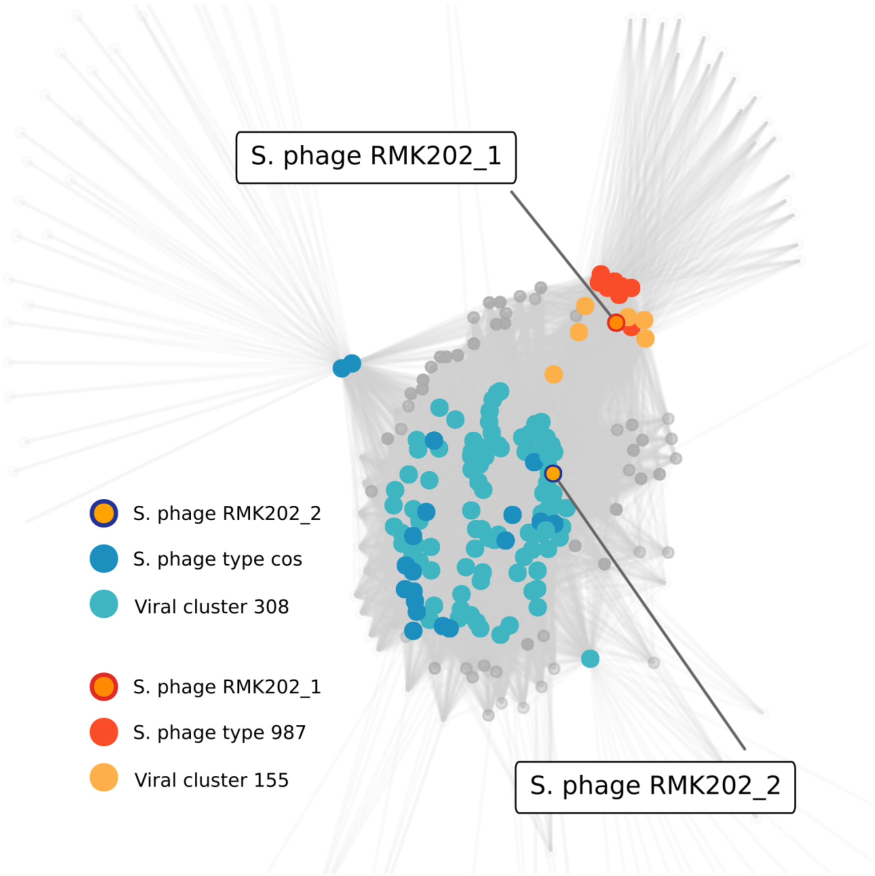

Figure 24. Similarity network of the two assembled *Streptococcus* phage genomes with previously sequenced phages and viral contigs based on vCONTACT v2.

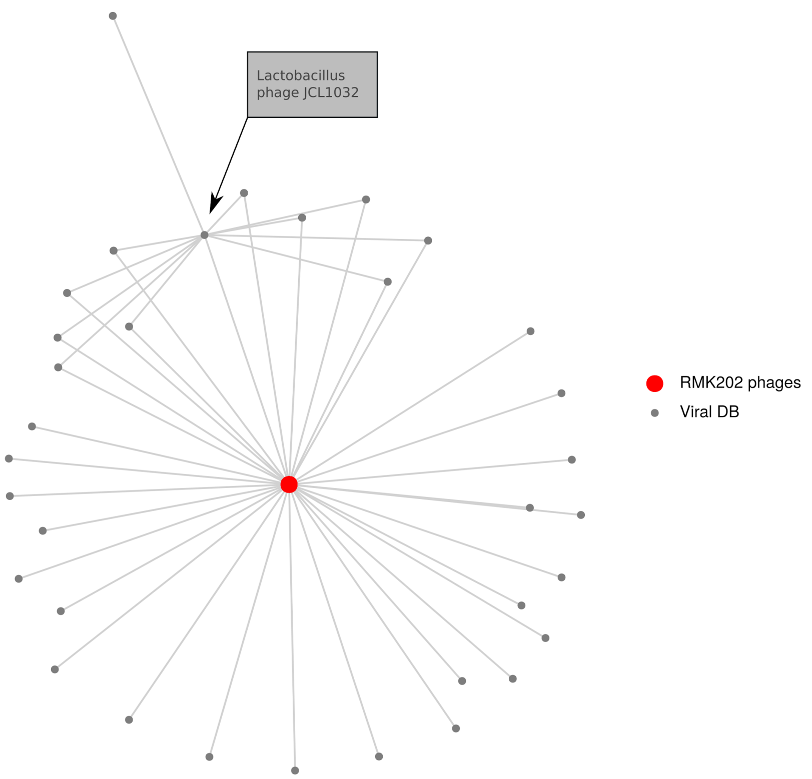

Figure 25. This plot will contain the *L. delbrueckii* phage network including the closest phage hit *Lactobacillus* phage JCL1032.

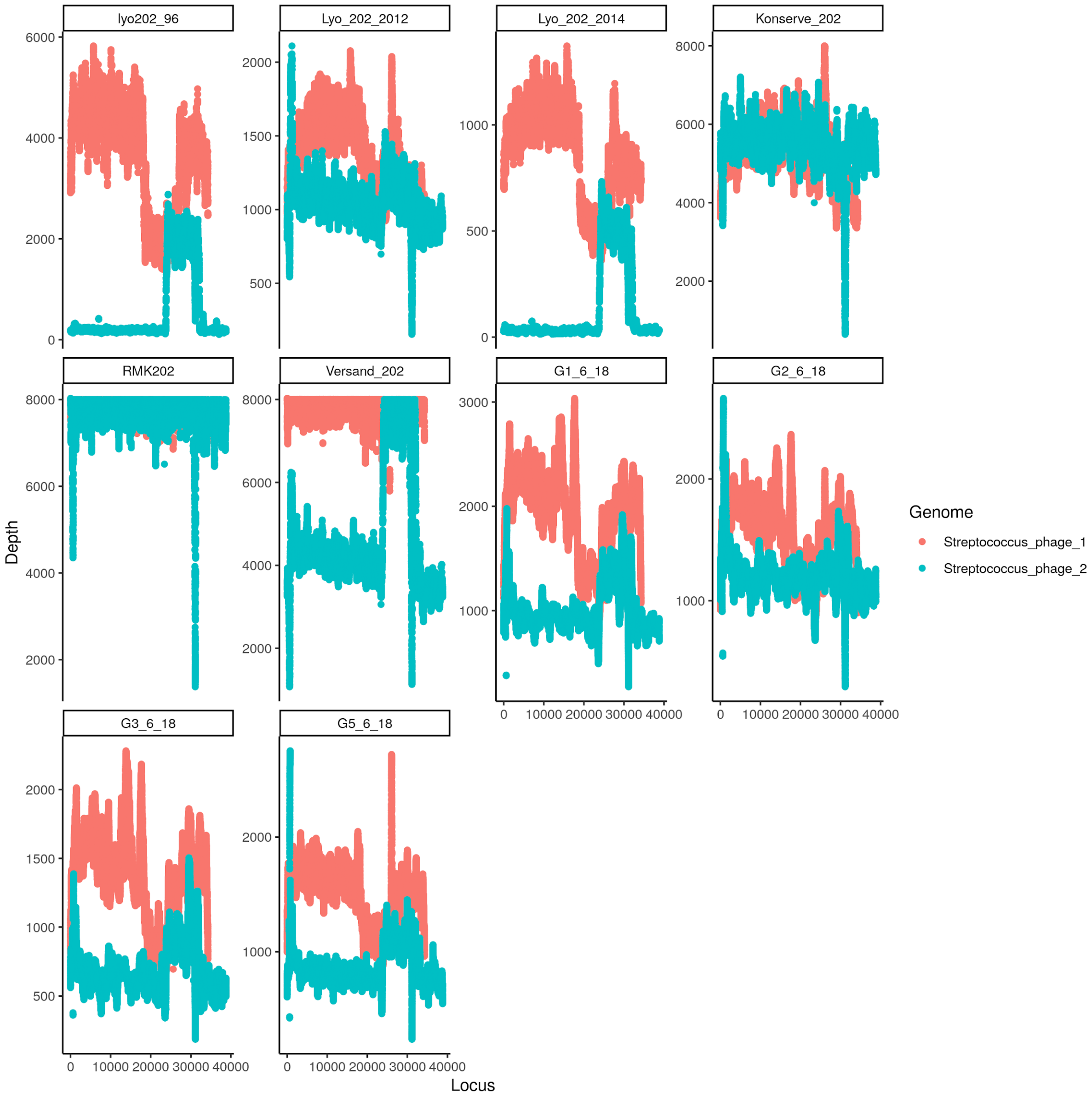

Figure 26. *Streptococcus* phage coverage in all metagenomic samples. The two genomes are very similar, especially in the lysis and lysogenic region, we thus see similar read recruitment by the different genomes. Note, the lysis & lysogenic region that is shared among the two phage genomes is recruiting the reads randomly to one or the other genome.

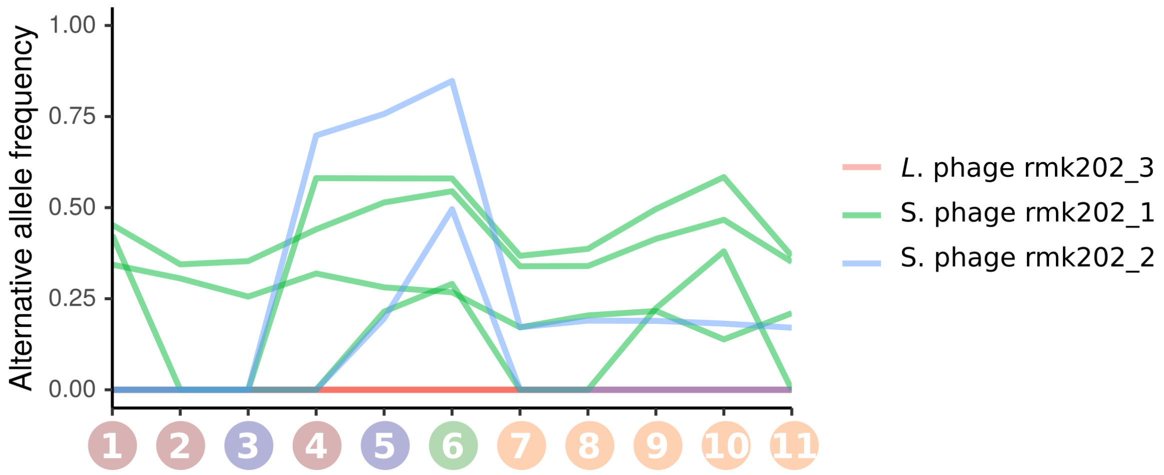

Figure 27. Alternative allele frequencies of the SNVs from the different phages. (The legend for the samples on the x-axis are illustrated in Fig. 2A).

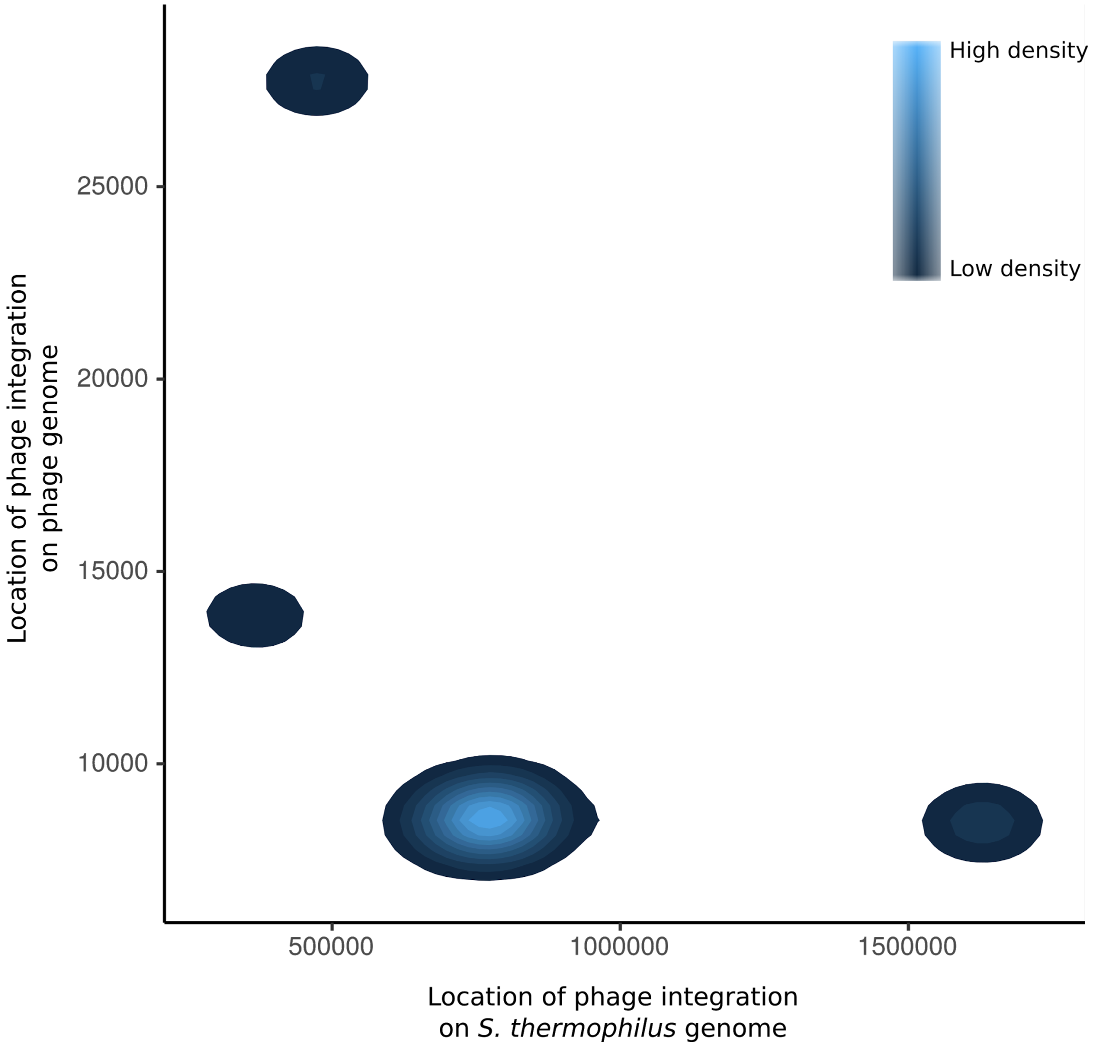

Figure 28. Four different putative integration sites were identified in the *S. thermophilus* (x-axis) and the phage genome (y-axis). The densities illustrate the number of reads mapping to the different locations.

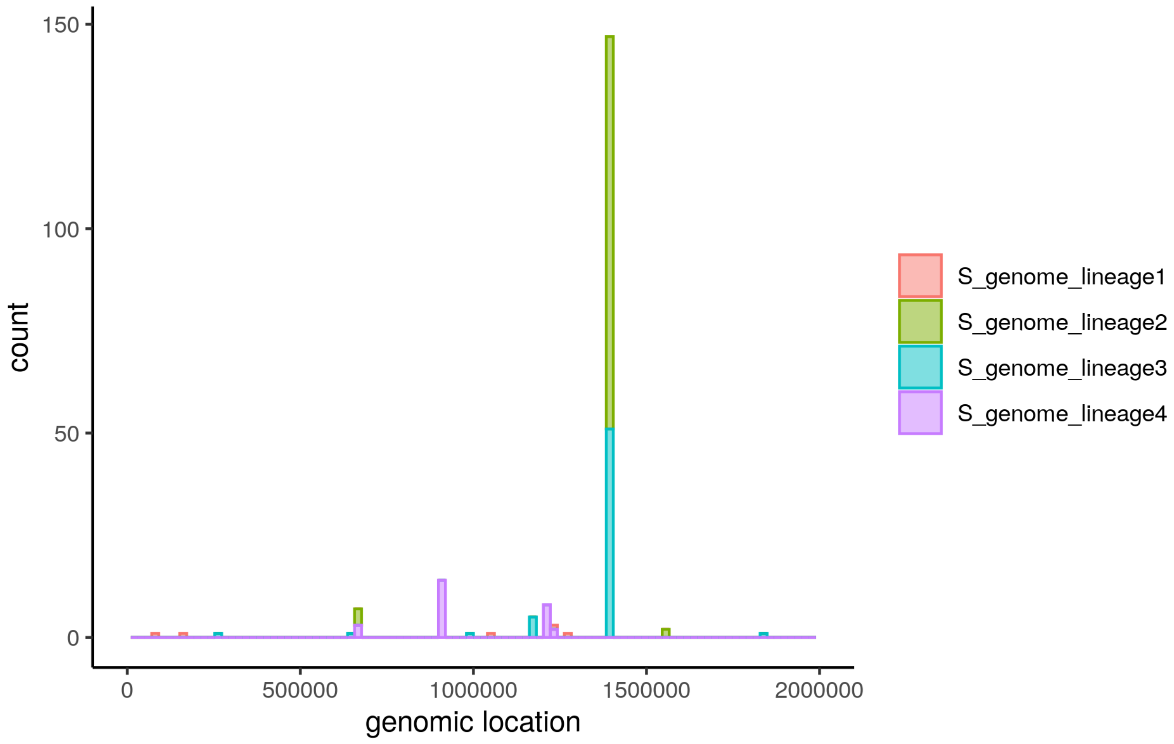

Figure 29. Genomic location on *S. thermophilus* where the lineage specific mate pair reads that span bacterial and phage genomes.

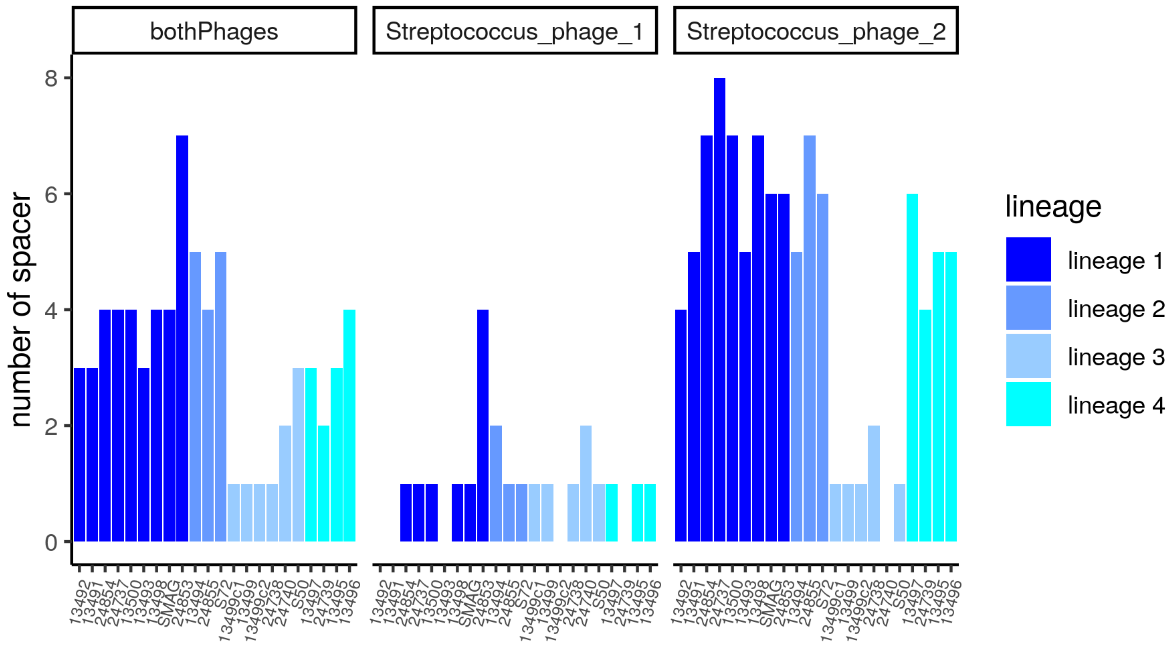

Figure 30. Number of CRISPR spacers that map to the two *Streptococcus* phages present in the starter culture. The color code for the genomes is illustrated in the legend and corresponds to the phylogenetic lineage.

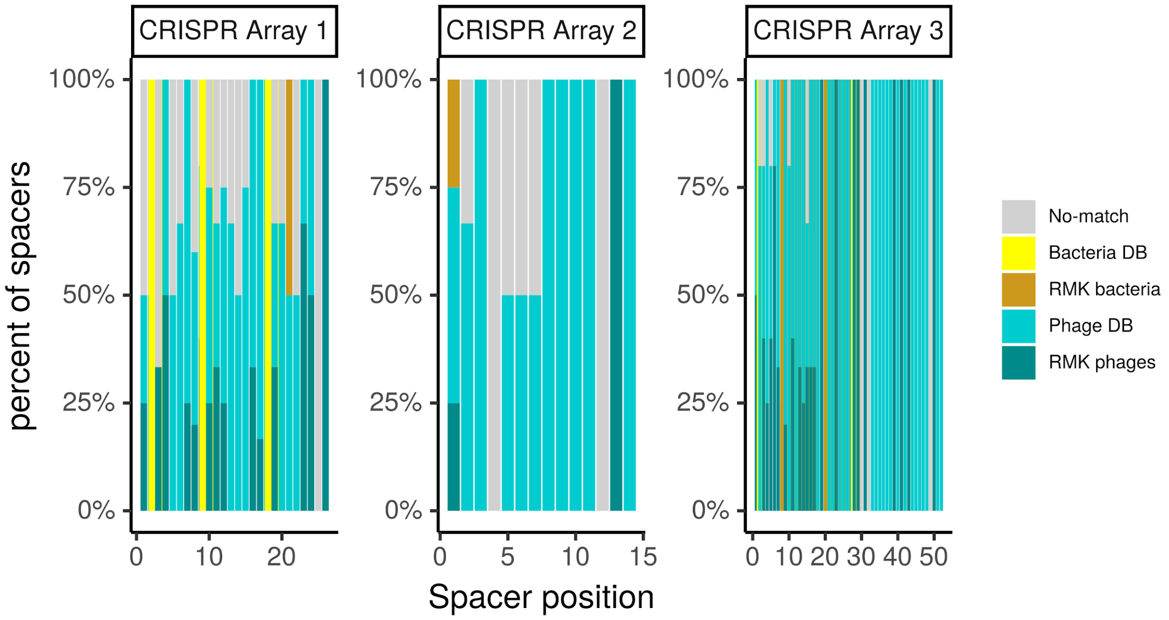

Figure 31. Origin of the CRISPR spacers plot according to the location on the CRISPR array.

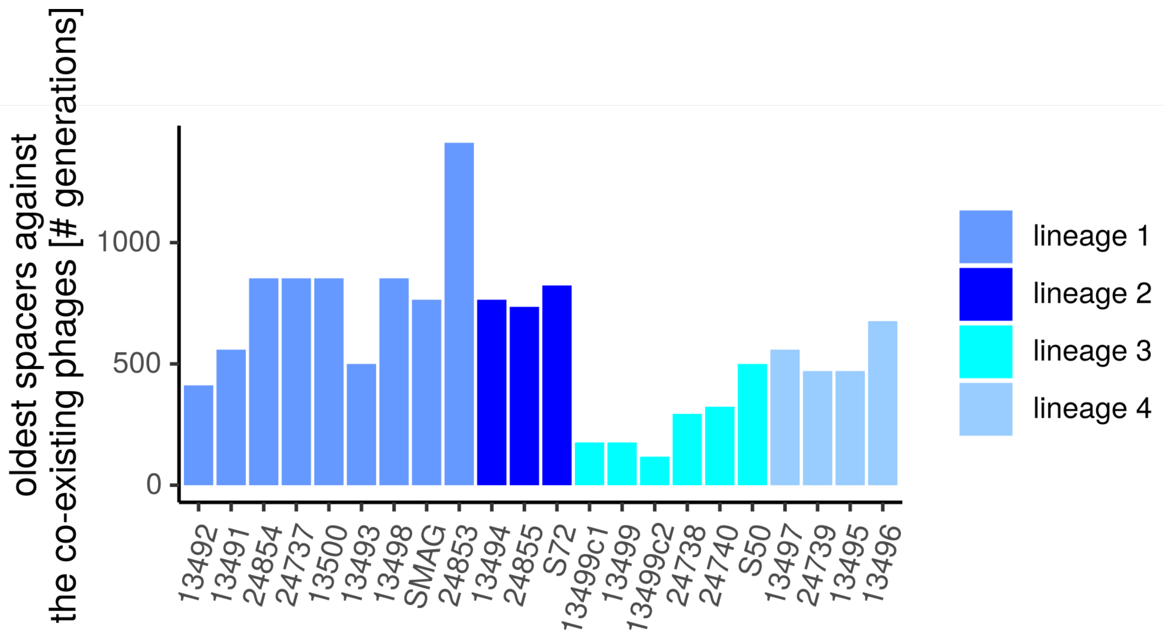

Figure 32. The estimated age (measured in generations) of the oldest spacers mapping against the co-existing phages for all *S. thermophilus* genomes. The number of generations was calculated by including the previously observed 0.024 spacer per generation turnover rate.

References

Fuchsmann, Pascal, Mireille Tena Stern, Patrick Bischoff, René Badertscher, Katharina Breme, and Barbara Walther. 2019. “Development and Performance Evaluation of a Novel Dynamic Headspace Vacuum Transfer ‘In Trap’ Extraction Method for Volatile Compounds and Comparison with Headspace Solid-Phase Microextraction and Headspace in-Tube Extraction.” *Journal of Chromatography A*. https://doi.org/10.1016/j.chroma.2019.05.016.

Vingataramin, Laurie, and Eric H. Frost. 2015. “A Single Protocol for Extraction of gDNA from Bacteria and Yeast.” *BioTechniques* 58 (3): 120–25.

Wick, Ryan R., Mark B. Schultz, Justin Zobel, and Kathryn E. Holt. 2015. “Bandage: Interactive Visualization of de Novo Genome Assemblies.” *Bioinformatics* 31 (20): 3350–52.

Borshchevskaya, L. N., T. L. Gordeeva, A. N. Kalinina, and S. P. Sineokii. 2016. “Spectrophotometric Determination of Lactic Acid.” Journal of Analytical Chemistry. https://doi.org/10.1134/s1061934816080037.

Danecek, P., A. Auton, G. Abecasis, C. A. Albers, E. Banks, M. A. DePristo, R. E. Handsaker, et al. 2011. “The Variant Call Format and VCFtools.” Bioinformatics. https://doi.org/10.1093/bioinformatics/btr330.

De Baets, Greet, Joost Van Durme, Joke Reumers, Sebastian Maurer-Stroh, Peter Vanhee, Joaquin Dopazo, Joost Schymkowitz, and Frederic Rousseau. 2012. “SNPeffect 4.0: On-Line Prediction of Molecular and Structural Effects of Protein-Coding Variants.” Nucleic Acids Research 40 (Database issue): D935–39.

Ellegaard, Kirsten M., and Philipp Engel. 2019. “Genomic Diversity Landscape of the Honey Bee Gut Microbiota.” Nature Communications 10 (1): 446.

Garrison, Erik, and Gabor Marth. 2012. “Haplotype-Based Variant Detection from Short-Read Sequencing.” arXiv.org, July. https://doi.org/arXiv:1207.3907.

Huerta-Cepas, Jaime, Damian Szklarczyk, Davide Heller, Ana Hernández-Plaza, Sofia K. Forslund, Helen Cook, Daniel R. Mende, et al. 2019. “eggNOG 5.0: A Hierarchical, Functionally and Phylogenetically Annotated Orthology Resource Based on 5090 Organisms and 2502 Viruses.” Nucleic Acids Research 47 (D1): D309–14.

Jain, Chirag, Luis M. Rodriguez-R, Adam M. Phillippy, Konstantinos T. Konstantinidis, and Srinivas Aluru. 2018. “High Throughput ANI Analysis of 90K Prokaryotic Genomes Reveals Clear Species Boundaries.” Nature Communications 9 (1): 5114.

Kolmogorov, Mikhail, Jeffrey Yuan, Yu Lin, and Pavel A. Pevzner. 2019. “Assembly of Long, Error-Prone Reads Using Repeat Graphs.” Nature Biotechnology 37 (5): 540–46.

Krzywinski, Martin, Jacqueline Schein, Inanç Birol, Joseph Connors, Randy Gascoyne, Doug Horsman, Steven J. Jones, and Marco A. Marra. 2009. “Circos: An Information Aesthetic for Comparative Genomics.” Genome Research 19 (9): 1639–45.

Li, Heng, and Richard Durbin. 2010. “Fast and Accurate Long-Read Alignment with Burrows-Wheeler Transform.” Bioinformatics 26 (5): 589–95.

Martin, Marcel. 2011. “Cutadapt Removes Adapter Sequences from High-Throughput Sequencing Reads.” EMBnet.journal 17 (1): 10–12.

Mikheenko, Alla, Vladislav Saveliev, and Alexey Gurevich. 2016. “MetaQUAST: Evaluation of Metagenome Assemblies.” Bioinformatics 32 (7): 1088–90.

Milanese, Alessio, Daniel R. Mende, Lucas Paoli, Guillem Salazar, Hans-Joachim Ruscheweyh, Miguelangel Cuenca, Pascal Hingamp, et al. 2019. “Microbial Abundance, Activity and Population Genomic Profiling with mOTUs2.” Nature Communications 10 (1): 1014.

Nurk, Sergey, Anton Bankevich, Dmitry Antipov, Alexey Gurevich, Anton Korobeynikov, Alla Lapidus, Andrey Prjibelsky, et al. 2013. “Assembling Genomes and Mini-Metagenomes from Highly Chimeric Reads.” Lecture Notes in Computer Science.

https://doi.org/10.1007/978-3-642-37195-0_13.
Shani, Noam, Dino Isolini, Daniel Marzohl, and Hélène Berthoud. 2021. “Evaluation of a New Culture Medium for the Enumeration and Isolation of Streptococcus Salivarius Subsp. Thermophilus from Cheese.” Food Microbiology. https://doi.org/10.1016/j.fm.2020.103672.
Simão, Felipe A., Robert M. Waterhouse, Panagiotis Ioannidis, Evgenia V. Kriventseva, and Evgeny M. Zdobnov. 2015. “BUSCO: Assessing Genome Assembly and Annotation Completeness with Single-Copy Orthologs.” Bioinformatics 31 (19): 3210–12.
Sjöqvist, C., L. F. Delgado Zambrano, J. Alneberg, and A. F. Andersson. n.d. “Revealing Ecologically Coherent Population Structure of Uncultivated Bacterioplankton with POGENOM.” https://doi.org/10.1101/2020.03.25.999755.
Tatusova, Tatiana, Michael DiCuccio, Azat Badretdin, Vyacheslav Chetvernin, Eric P. Nawrocki, Leonid Zaslavsky, Alexandre Lomsadze, Kim D. Pruitt, Mark Borodovsky, and James Ostell. 2016. “NCBI Prokaryotic Genome Annotation Pipeline.” Nucleic Acids Research. https://doi.org/10.1093/nar/gkw569.
Tonkin-Hill, Gerry, Neil MacAlasdair, Christopher Ruis, Aaron Weimann, Gal Horesh, John A. Lees, Rebecca A. Gladstone, et al. 2020. “Producing Polished Prokaryotic Pangenomes with the Panaroo Pipeline.” Genome Biology 21 (1): 180.
Vaser, Robert, Ivan Sović, Niranjan Nagarajan, and Mile Šikić. 2017. “Fast and Accurate de Novo Genome Assembly from Long Uncorrected Reads.” Genome Research. https://doi.org/10.1101/gr.214270.116.
Vingataramin, Laurie, and Eric H. Frost. 2015. “A Single Protocol for Extraction of gDNA from Bacteria and Yeast.” BioTechniques 58 (3): 120–25.
