# Supplementary material for: Functional strain redundancy and persistent phage infection in Swiss hard cheese starter cultures
Source: ISME J. 2021 Aug 6;16(2):388–99. doi: 10.1038/s41396-021-01071-0 (PMC8776748; doi:10.1038/s41396-021-01071-0)
Supplement: Supplementary file 2 — SUPPLEMENTARY_TABLE_1 [file 41396_2021_1071_MOESM2_ESM.pdf]

Supplement: Functional strain redundancy and persistent phage infection in Swiss hard cheese starter cultures

Table1: Statistics of all assembled or used genomes. GC stands for Guanine and Cytosine percentage in the genome. CRISPR stands for the number of CRISPR arrays annotated. BUSCO stands for the percent Busco completeness predicted.

Species	Strain	Technology	# Contigs	Genome size	GC	Prophage	Plasmid	genes	Pseudogenes	rRNA	tRNA	transposase	CRISPR	BUSCO
S. thermophilus	13494	Nanopore&I.	2	1882333	39	0	1	1946	230	88	67	79	3	99
S. thermophilus	13496	Nanopore&I.	1	1895875	39	0	0	1980	232	89	67	105	2	99
S. thermophilus	13498	Nanopore&I.	1	1865049	39	0	0	1934	223	88	66	81	3	99
S. thermophilus	13499	Nanopore&I.	1	1885735	39	0	0	1976	237	89	67	99	3	98
S. thermophilus	24737	Nanopore&I.	2	1868869	39	0	1	1938	229	91	68	81	3	99
S. thermophilus	24738	Nanopore&I.	1	1885982	39	0	0	1983	248	88	66	99	3	98
S. thermophilus	24739	Nanopore&I.	1	1934999	39	1	0	2027	234	89	67	103	2	99
S. thermophilus	24740	Nanopore&I.	1	1880058	39	0	0	1973	247	76	57	98	3	99
S. thermophilus	24853	Nanopore&I.	1	1847542	39	0	0	1920	221	89	67	73	3	99
S. thermophilus	24854	Nanopore&I.	2	1869650	39	0	1	1939	225	92	68	81	3	99
S. thermophilus	24855	Nanopore&I.	2	1926384	39	0	1	2010	261	110	85	85	3	99
S. thermophilus	13491	Illumina	46	1838081	39	0	0	1943	233	74	62	92	3	99
S. thermophilus	13492	Illumina	40	1835354	39	0	0	1923	229	59	50	89	3	99
S. thermophilus	13493	Illumina	54	1836485	39	0	0	1950	236	69	58	101	3	99
S. thermophilus	13495	Illumina	104	1803669	39	0	0	1937	251	59	50	91	2	95
S. thermophilus	13497	Illumina	63	1834883	39	0	0	1944	238	59	49	100	2	98
S. thermophilus	13499c1	Illumina	58	1838913	39	0	0	1960	248	63	53	100	3	99
S. thermophilus	13499c2	Illumina	60	1839354	39	0	0	1965	249	63	53	104	3	99
S. thermophilus	13500	Illumina	40	1831933	39	0	0	1938	230	74	62	87	3	99
S. thermophilus	S50	Illumina	43	1842362	39	0	0	1921	231	47	42	74	3	98
S. thermophilus	S72	Illumina	31	1835142	39	0	0	1897	225	49	43	68	3	99
S. thermophilus	SMAG	MAG	1	1879576	39	1	0	1975	227	89	67	75	3	99
S. thermophilus	19258	Typestrain	1	2102268	39	0	0	2230	287	75	56	100	2	99
L. delbrueckii	11141	Illumina	155	1994221	49	0	0	2112	191	96	80	199	3	97
L. delbrueckii	11142	Illumina	159	2017445	49	0	1	2140	170	96	79	215	2	98
L. delbrueckii	11143	Illumina	164	2002804	49	0	1	2142	185	91	73	225	3	97
L. delbrueckii	12104	Illumina	165	2003390	49	0	0	2168	197	100	80	247	3	97
L. delbrueckii	12105	Illumina	162	2012792	49	0	1	2170	196	99	81	241	3	98
L. delbrueckii	12107	Illumina	166	2007880	49	0	1	2129	180	104	82	202	3	98
L. delbrueckii	12109	Illumina	162	1985627	49	0	1	2079	187	88	70	171	3	98
L. delbrueckii	24776	Illumina	175	1954721	49	0	1	2041	172	98	78	126	3	97
L. delbrueckii	24777	Illumina	168	1933884	49	0	1	2011	166	94	77	120	3	97
L. delbrueckii	24778	Illumina	166	1910523	50	0	1	1920	153	62	54	48	3	97
L. delbrueckii	24779	Illumina	171	1955208	49	0	1	2032	164	91	74	125	3	97
L. delbrueckii	24780	Illumina	175	1953065	49	0	1	2035	170	92	74	131	3	97
L. delbrueckii	24781	Illumina	171	1943061	49	0	0	2021	163	92	74	126	3	97
L. delbrueckii	24782	Illumina	173	1888663	50	0	0	1974	162	95	77	126	1	97
L. delbrueckii	24783	Illumina	176	1946687	49	0	0	2020	167	94	77	122	3	97
L. delbrueckii	24798	Illumina	171	1956412	49	0	1	2032	172	92	77	127	3	97
L. delbrueckii	LMAG	MAG	1	2184491	49	0	1	2163	240	125	95	212	4	98
L. delbrueckii	20072	Typestrain	1	2165984	49	0	0	2152	228	124	94	210	1	98
