# Supplementary material for: Functional strain redundancy and persistent phage infection in Swiss hard cheese starter cultures
Source: ISME J. 2021 Aug 6;16(2):388–99. doi: 10.1038/s41396-021-01071-0 (PMC8776748; doi:10.1038/s41396-021-01071-0)
Supplement: Supplementary file 3 — SUPPLEMENTARY_TABLE_2 [file 41396_2021_1071_MOESM3_ESM.pdf]

Streptococcus_phage_1	Prodigal_v2.6.3	CDS	5	487	81.7	+	0
Streptococcus_phage_1	Prodigal_v2.6.3	CDS	549	743	16.4	+	0
Streptococcus_phage_1	Prodigal_v2.6.3	CDS	789	1952	224.2	+	0
Streptococcus_phage_1	Prodigal_v2.6.3	CDS	1953	3293	268.8	+	0
Streptococcus_phage_1	Prodigal_v2.6.3	CDS	3297	4340	203.6	+	0
Streptococcus_phage_1	Prodigal_v2.6.3	CDS	4457	5056	146.7	+	0
Streptococcus_phage_1	Prodigal_v2.6.3	CDS	5072	5935	187.1	+	0
Streptococcus_phage_1	Prodigal_v2.6.3	CDS	6001	6192	11.2	+	0
Streptococcus_phage_1	Prodigal_v2.6.3	CDS	6206	6538	67.8	+	0
Streptococcus_phage_1	Prodigal_v2.6.3	CDS	6535	6846	56.4	+	0
Streptococcus_phage_1	Prodigal_v2.6.3	CDS	6843	7169	66	+	0
Streptococcus_phage_1	Prodigal_v2.6.3	CDS	7166	7555	63.2	+	0
Streptococcus_phage_1	Prodigal_v2.6.3	CDS	7566	8063	106.3	+	0
Streptococcus_phage_1	Prodigal_v2.6.3	CDS	8179	8529	69.6	+	0
Streptococcus_phage_1	Prodigal_v2.6.3	CDS	8571	8882	39.9	+	0
Streptococcus_phage_1	Prodigal_v2.6.3	CDS	8898	11111	411.1	+	0
Streptococcus_phage_1	Prodigal_v2.6.3	CDS	11121	11882	141.8	+	0
Streptococcus_phage_1	Prodigal_v2.6.3	CDS	11882	14614	526.8	+	0
Streptococcus_phage_1	Prodigal_v2.6.3	CDS	14626	16569	327.4	+	0
Streptococcus_phage_1	Prodigal_v2.6.3	CDS	16734	17150	71.2	+	0
Streptococcus_phage_1	Prodigal_v2.6.3	CDS	17167	17409	51.3	+	0
Streptococcus_phage_1	Prodigal_v2.6.3	CDS	17411	18256	168.1	+	0
Streptococcus_phage_1	Prodigal_v2.6.3	CDS	18709	19044	93.4	+	0
Streptococcus_phage_1	Prodigal_v2.6.3	CDS	19070	19360	56.7	+	0
Streptococcus_phage_1	Prodigal_v2.6.3	CDS	19389	19940	106.1	+	0
Streptococcus_phage_1	Prodigal_v2.6.3	CDS	20063	20461	94.4	+	0
Streptococcus_phage_1	Prodigal_v2.6.3	CDS	20532	20954	86.7	+	0
Streptococcus_phage_1	Prodigal_v2.6.3	CDS	21141	22211	185.3	-	0
Streptococcus_phage_1	Prodigal_v2.6.3	CDS	22282	22995	48	-	0
Streptococcus_phage_1	Prodigal_v2.6.3	CDS	23046	23432	58.9	-	0
Streptococcus_phage_1	Prodigal_v2.6.3	CDS	23422	23826	64.3	-	0
Streptococcus_phage_1	Prodigal_v2.6.3	CDS	23975	24214	1.8	+	0
Streptococcus_phage_1	Prodigal_v2.6.3	CDS	24226	24981	145.1	+	0
Streptococcus_phage_1	Prodigal_v2.6.3	CDS	24993	25274	55.8	+	0
Streptococcus_phage_1	Prodigal_v2.6.3	CDS	25652	26485	162	+	0
Streptococcus_phage_1	Prodigal_v2.6.3	CDS	26498	27280	126.1	+	0
Streptococcus_phage_1	Prodigal_v2.6.3	CDS	27277	27459	28.7	+	0
Streptococcus_phage_1	Prodigal_v2.6.3	CDS	27615	28277	141.5	+	0
Streptococcus_phage_1	Prodigal_v2.6.3	CDS	28280	29239	213.6	+	0
Streptococcus_phage_1	Prodigal_v2.6.3	CDS	29260	29706	86.9	+	0
Streptococcus_phage_1	Prodigal_v2.6.3	CDS	29721	30176	63	+	0
Streptococcus_phage_1	Prodigal_v2.6.3	CDS	30173	30409	32.5	+	0
Streptococcus_phage_1	Prodigal_v2.6.3	CDS	30400	30573	20.7	+	0
Streptococcus_phage_1	Prodigal_v2.6.3	CDS	30611	30766	26.9	+	0
Streptococcus_phage_1	Prodigal_v2.6.3	CDS	30891	31274	50.1	+	0
Streptococcus_phage_1	Prodigal_v2.6.3	CDS	31287	31790	93.9	+	0
Streptococcus_phage_1	Prodigal_v2.6.3	CDS	31791	32303	107.1	+	0
Streptococcus_phage_1	Prodigal_v2.6.3	CDS	32272	32580	55.3	+	0
Streptococcus_phage_1	Prodigal_v2.6.3	CDS	32577	33284	135.1	+	0
Streptococcus_phage_1	Prodigal_v2.6.3	CDS	33649	34098	73.5	+	0

ID=1_1;name=hypothetical protein rmk202_01_49
ID=1_2;name=small terminase subunit
ID=1_3;name=large terminase subunit
ID=1_4;name=portal protein
ID=1_5;name=minor capsid protein
ID=1_6;name=scaffolding protein
ID=1_7;name=major capsid protein
ID=1_8;name=hypothetical protein rmk202_01_08
ID=1_9;name=head-tail connector protein
ID=1_10;name=Head-closure protein
ID=1_11;name=tail protein
ID=1_12;name=tail completion protein
ID=1_13;name=major tail protein
ID=1_14;name=tail chaperone protein
ID=1_15;name=hypothetical protein rmk202_01_15
ID=1_16;name=tape-measure protein
ID=1_17;name=distal tail protein
ID=1_18;name=tail-associated lysin
ID=1_19;name=upper baseplate protein
ID=1_20;name=transcription termination factor
ID=1_21;name=holin
ID=1_22;name=lysin
ID=1_23;name=hypothetical protein rmk202_01_23
ID=1_24;name=hypothetical protein rmk202_01_24
ID=1_25;name=Acr-like protein
ID=1_26;name=AcrIIA3-like protein
ID=1_27;name=hypothetical protein rmk202_01_27
ID=1_28;name=Integrase
ID=1_29;name=putative protease
ID=1_30;name=ImmA/IrrE family metallo-endopeptidase
ID=1_31;name=putative repressor
ID=1_32;name=transcriptional regulator
ID=1_33;name=antirepressor protein
ID=1_34;name=DNA-binding protein
ID=1_35;name=replication initiation
ID=1_36;name=helicase loader
ID=1_37;name=hypothetical protein rmk202_01_37
ID=1_38;name=Erf protein
ID=1_39;name=hypothetical protein rmk202_01_39
ID=1_40;name=single-stranded DNA-binding protein
ID=1_41;name=holliday junction resolvase
ID=1_42;name=hypothetical protein rmk202_01_42
ID=1_43;name=hypothetical protein rmk202_01_43
ID=1_44;name=hypothetical protein rmk202_01_44
ID=1_45;name=hypothetical protein rmk202_01_45
ID=1_46;name=hypothetical protein rmk202_01_46
ID=1_47;name=DNA-binding protein
ID=1_48;name=hypothetical protein rmk202_01_48
ID=1_49;name=hypothetical protein rmk202_01_49
ID=1_50;name=ArpU late transcriptional regulator

Streptococcus_phage_2	Prodigal_v2.6.3	CDS	3	134	7.7	+	0
Streptococcus_phage_2	Prodigal_v2.6.3	CDS	88	522	43.7	+	0
Streptococcus_phage_2	Prodigal_v2.6.3	CDS	703	1164	86	+	0
Streptococcus_phage_2	Prodigal_v2.6.3	CDS	1177	3048	312.5	+	0
Streptococcus_phage_2	Prodigal_v2.6.3	CDS	3006	3170	4.6	+	0
Streptococcus_phage_2	Prodigal_v2.6.3	CDS	3249	4409	200.8	+	0
Streptococcus_phage_2	Prodigal_v2.6.3	CDS	4396	5064	141.7	+	0
Streptococcus_phage_2	Prodigal_v2.6.3	CDS	5079	6272	277.2	+	0
Streptococcus_phage_2	Prodigal_v2.6.3	CDS	6287	6601	11	+	0
Streptococcus_phage_2	Prodigal_v2.6.3	CDS	6601	6951	60.5	+	0
Streptococcus_phage_2	Prodigal_v2.6.3	CDS	6958	7383	70.3	+	0
Streptococcus_phage_2	Prodigal_v2.6.3	CDS	7384	7755	68.9	+	0
Streptococcus_phage_2	Prodigal_v2.6.3	CDS	7777	8388	151.6	+	0
Streptococcus_phage_2	Prodigal_v2.6.3	CDS	8464	8817	89.9	+	0
Streptococcus_phage_2	Prodigal_v2.6.3	CDS	9036	13847	980.6	+	0
Streptococcus_phage_2	Prodigal_v2.6.3	CDS	13844	15409	255.5	+	0
Streptococcus_phage_2	Prodigal_v2.6.3	CDS	15409	19329	770.4	+	0
Streptococcus_phage_2	Prodigal_v2.6.3	CDS	19332	21407	340.9	+	0
Streptococcus_phage_2	Prodigal_v2.6.3	CDS	21428	21823	86.8	+	0
Streptococcus_phage_2	Prodigal_v2.6.3	CDS	21830	21976	25.2	+	0
Streptococcus_phage_2	Prodigal_v2.6.3	CDS	21990	22313	64.5	+	0
Streptococcus_phage_2	Prodigal_v2.6.3	CDS	22322	22564	38.1	+	0
Streptococcus_phage_2	Prodigal_v2.6.3	CDS	22566	23411	173.7	+	0
Streptococcus_phage_2	Prodigal_v2.6.3	CDS	23729	24064	92.5	+	0
Streptococcus_phage_2	Prodigal_v2.6.3	CDS	24090	24380	59.7	+	0
Streptococcus_phage_2	Prodigal_v2.6.3	CDS	24409	24960	109.6	+	0
Streptococcus_phage_2	Prodigal_v2.6.3	CDS	25083	25481	93.9	+	0
Streptococcus_phage_2	Prodigal_v2.6.3	CDS	25552	25974	82.9	+	0
Streptococcus_phage_2	Prodigal_v2.6.3	CDS	26161	27231	159.6	-	0
Streptococcus_phage_2	Prodigal_v2.6.3	CDS	27302	28015	68.7	-	0
Streptococcus_phage_2	Prodigal_v2.6.3	CDS	28066	28452	57.3	-	0
Streptococcus_phage_2	Prodigal_v2.6.3	CDS	28442	28846	55.6	-	0
Streptococcus_phage_2	Prodigal_v2.6.3	CDS	28995	29234	17.8	+	0
Streptococcus_phage_2	Prodigal_v2.6.3	CDS	29246	30001	135.9	+	0
Streptococcus_phage_2	Prodigal_v2.6.3	CDS	30013	30294	44.7	+	0
Streptococcus_phage_2	Prodigal_v2.6.3	CDS	30672	31517	142.3	+	0
Streptococcus_phage_2	Prodigal_v2.6.3	CDS	31530	32312	124.4	+	0
Streptococcus_phage_2	Prodigal_v2.6.3	CDS	32309	32491	25.7	+	0
Streptococcus_phage_2	Prodigal_v2.6.3	CDS	32648	33307	131.5	+	0
Streptococcus_phage_2	Prodigal_v2.6.3	CDS	33310	34281	209.1	+	0
Streptococcus_phage_2	Prodigal_v2.6.3	CDS	34281	34730	96.1	+	0
Streptococcus_phage_2	Prodigal_v2.6.3	CDS	34740	35201	68.2	+	0
Streptococcus_phage_2	Prodigal_v2.6.3	CDS	35289	35447	25.8	+	0
Streptococcus_phage_2	Prodigal_v2.6.3	CDS	35440	36192	132.1	+	0
Streptococcus_phage_2	Prodigal_v2.6.3	CDS	36269	36652	57	+	0
Streptococcus_phage_2	Prodigal_v2.6.3	CDS	36665	37015	77.7	+	0
Streptococcus_phage_2	Prodigal_v2.6.3	CDS	36984	37292	36.4	+	0
Streptococcus_phage_2	Prodigal_v2.6.3	CDS	37289	37996	131.7	+	0
Streptococcus_phage_2	Prodigal_v2.6.3	CDS	38288	38686	68.8	+	0

ID=1_1;name=HNH endonuclease
ID=1_2;name=terminase small subunit
ID=1_3;name=large terminase subunit
ID=1_4;name=hypothetical protein rmk202_02_04
ID=1_5;name=portal protein
ID=1_6;name=putative scaffolding protein
ID=1_7;name=major capsid protein
ID=1_8;name=head-tail connector protein
ID=1_9;name=Head-closure protein
ID=1_10;name=tail protein
ID=1_11;name=tail completion protein
ID=1_12;name=major tail protein
ID=1_13;name=tail protein
ID=1_14;name=tape measure protein
ID=1_15;name=distal tail protein
ID=1_16;name=capsid and scaffold protein
ID=1_17;name=baseplate component
ID=1_18;name=hypothetical protein rmk202_02_18
ID=1_19;name=hypothetical protein rmk202_02_19
ID=1_20;name=hypothetical protein rmk202_02_20
ID=1_21;name=holin
ID=1_22;name=lysin
ID=1_23;name=hypothetical protein rmk202_02_23
ID=1_24;name=hypothetical protein rmk202_02_24
ID=1_25;name=Acr-like protein
ID=1_26;name=AcrIIA3-like protein
ID=1_27;name=hypothetical protein rmk202_02_27
ID=1_28;name=Integrase
ID=1_29;name=putative protease
ID=1_30;name=ImmA/IrrE family metallo-endopeptidase
ID=1_31;name=putative repressor
ID=1_32;name=transcriptional regulator
ID=1_33;name=antirepressor protein
ID=1_34;name=hypothetical protein rmk202_02_34
ID=1_35;name=replication initiation
ID=1_36;name=helicase loader
ID=1_37;name=hypothetical protein rmk202_02_37
ID=1_38;name=single-stranded DNA-binding protein
ID=1_39;name=hypothetical protein rmk202_02_39
ID=1_40;name=single-stranded DNA-binding protein
ID=1_41;name=endodeoxyribonuclease RusA
ID=1_42;name=hypothetical protein rmk202_02_42
ID=1_43;name=adenine-specific methyltransferase
ID=1_44;name=hypothetical protein rmk202_02_44
ID=1_45;name=DNA-binding protein
ID=1_46;name=hypothetical protein rmk202_02_46
ID=1_47;name=hypothetical protein rmk202_02_47
ID=1_48;name=late transcriptional regulator
ID=1_49;name=hypothetical protein rmk202_02_49
