# Supplementary material for: Functional strain redundancy and persistent phage infection in Swiss hard cheese starter cultures
Source: ISME J. 2021 Aug 6;16(2):388–99. doi: 10.1038/s41396-021-01071-0 (PMC8776748; doi:10.1038/s41396-021-01071-0)
Supplement: Supplementary file 4 — SUPPLEMENTARY_METHODS [file 41396_2021_1071_MOESM4_ESM.pdf]

Supplement methods: Functional strain redundancy and persistent phage infection in Swiss hard cheese starter cultures

Genotyping

For genotyping, DNA from 100 µL of culture was extracted using the EtNa DNA isolation method (Vingataramin and Frost 2015) and diluted 10x before PCR amplification. Multiplex amplification was performed in 25 µL reactions containing 2.5 µL of GeneAmp® 10X PCR Buffer I containing 15 mM $MgCl_2$ (Thermo Fisher, Waltham, MA, United States), 0.9 mL $MgCl_2$ (50mM), 0.5 mL PCR nucleotide mix (10 mM; Promega AG, Dübendorf, Switzerland), 0.5 µL Q-Solution (Qiagen, Hombrechtikon, Switzerland), 2.5 µL primer mix (2 mM of each primer listed in SupplementTable1), 0.4 µL AmpliTaq Gold DNA polymerase (Thermo Fisher) and 2 µL of DNA.
After the initial heat activation at 95°C for 10 min followed 35 cycles at 94°C for 1 min, 58°C for 30 s, 72°C for 30 s, and a final extension at 72°C for 7 min. The amplification products were separated using the DNF-905-K0500 dsDNA 905 Reagent Kit (separation range 1-500 bp) on a Fragment Analyzer™ (Advanced Analytical Technologies, Ankeny, IA, USA) according to the manufacturer's instructions. The results were evaluated and compared with the PROSize software (V.3, Advanced Analytical Technologies).

GC-MS analysis

Samples for DHS-VTT-GC-MS analyses were prepared as follows: 250 mg of cheese starter culture were weighed in 20 mL headspace crimp glass vials. 25 µL of an internal standard solution composed by 0.5 ppm of paraldehyde, 0.25 ppm of tetradecane and 0.5 ppm of d4-δ-decalactone were added to the samples for analytical deviation correction.

Volatiles were extracted by dynamic headspace vacuum transfer in trap extraction (DHS-VTT) (Fuchsmann et al. 2019) and analyzed by gas-chromatography mass spectrometry (GC-MS). Volatile were adsorbed on a Tenax TA/Carbosieve III ITEX (in tube extraction) trap (BGB Analytik AG, Böckten, Switzerland) which was conditioned according to the supplier's temperature recommendations (320°C for 1 h) under a nitrogen stream of 100 mL min-1. During extraction, the syringe temperature was fixed at 100°C and the ITEX trap at 35°C. Samples were incubated for 10 min at 60°C and volatiles were extracted for 5 min at 5 mbar using a vacuum pump Buchi V-300 (Büchi, Flawil, Switzerland). After extraction, the sorbent and syringe were dried under a nitrogen stream for 5 and 20 min, respectively, at 220 mL min-1 to avoid injection of water in the column. Bound volatiles were desorbed for 2 min with a nitrogen flow of 100 mL min-1 at 240°C in a programmed temperature vaporizer (PTV) injector of type CIS4 (Gerstel AG, Sursee, Switzerland) in the vent mode at 50 mL min-1 and 0 kPa for 30 s. The injector containing a Tenax TA filled glass cooled to 10°C using liquid nitrogen trapped the compounds again. The volatiles were then released by heating the injector at a rate of 12°C sec-1 to 240°C. After injection, the trap was reconditioned according to the supplier's temperature recommendation (300°C) for 15 min under a nitrogen flow of 100 mL min-1.

The analyses were completed using an MPS2 autosampler (Gerstel AG) on an Agilent 7890B GC system coupled to an Agilent 5977B mass selective detector (MSD) (Agilent Technologies, Basel, Switzerland). Volatile compounds were separated on a OPTIMA

FFAPplus fused silica capillary column (polyethylene glycol nitroterephthalate, crosslinked, 60 m × 0.25 mm × 0.5 µm film; MACHEREY-NAGEL, Düren, Germany) with helium as the carrier gas at a constant flow of 1.5 mL min-1 (25.312 cm sec-1).
The oven temperature was programmed as follows: 5 min at 40°C, then heated to 240°C at a rate of 5°C min-1 with a final hold time of 20 min to make a total run time of 65 min.
The MS settings were as follows: transfer line at 250°C, source temperature at 230°C, and the analytes monitored in SCAN mode between 30 amu and 350 amu with 4 min solvent delay. The autosampler was controlled with a Cycle Composer V.1.5.4 (CTC Analytics, Zwingen, Switzerland) and PTV injector with Maestro1 software V.1.4.8.14/3.5 (Gerstel AG).

Peak extraction and grouping was obtained using MassHunter Profinder software version 10.0 (Agilent Technologies). A batch recursive feature extraction (small molecules/peptides) was performed on all data. For the molecular feature extraction (MFE), peaks with a height lower than 2000 counts were filtered out. Alignment parameters were set with a 0.30 min retention time tolerance and a 0.40 minimal dot-product value. A post-processing filter was set for the MFE with a score of minimum 50.0 with the requirement that a compound must satisfy the condition in at least one file across all sample files. For the Find by Ion parameters, a mass tolerance was set at ± 200 mDa and a retention time window of ± 0.30 min. Agile 2 integration was chosen and the chromatograms were smoothed using the Gaussian function with the function and Gaussian width both at 7 points. Spectra extraction was set to include spectra at apex of peak.

References

Fuchsmann, Pascal, Mireille Tena Stern, Patrick Bischoff, René Badertscher, Katharina Breme, and Barbara Walther. 2019. “Development and Performance Evaluation of a Novel Dynamic Headspace Vacuum Transfer ‘In Trap’ Extraction Method for Volatile Compounds and Comparison with Headspace Solid-Phase Microextraction and Headspace in-Tube Extraction.” *Journal of Chromatography A*. https://doi.org/10.1016/j.chroma.2019.05.016.

Vingataramin, Laurie, and Eric H. Frost. 2015. “A Single Protocol for Extraction of gDNA from Bacteria and Yeast.” *BioTechniques* 58 (3): 120–25.

Wick, Ryan R., Mark B. Schultz, Justin Zobel, and Kathryn E. Holt. 2015. “Bandage: Interactive Visualization of de Novo Genome Assemblies.” *Bioinformatics* 31 (20): 3350–52.

Borshchevskaya, L. N., T. L. Gordeeva, A. N. Kalinina, and S. P. Sineokii. 2016. “Spectrophotometric Determination of Lactic Acid.” Journal of Analytical Chemistry. https://doi.org/10.1134/s1061934816080037.

Danecek, P., A. Auton, G. Abecasis, C. A. Albers, E. Banks, M. A. DePristo, R. E. Handsaker, et al. 2011. “The Variant Call Format and VCFtools.” Bioinformatics. https://doi.org/10.1093/bioinformatics/btr330.

De Baets, Greet, Joost Van Durme, Joke Reumers, Sebastian Maurer-Stroh, Peter Vanhee, Joaquin Dopazo, Joost Schymkowitz, and Frederic Rousseau. 2012. “SNPeffect 4.0: On-Line Prediction of Molecular and Structural Effects of Protein-Coding Variants.” Nucleic Acids Research 40 (Database issue): D935–39.

Ellegaard, Kirsten M., and Philipp Engel. 2019. “Genomic Diversity Landscape of the Honey Bee Gut Microbiota.” Nature Communications 10 (1): 446.

Garrison, Erik, and Gabor Marth. 2012. “Haplotype-Based Variant Detection from Short-Read Sequencing.” arXiv.org, July. https://doi.org/arXiv:1207.3907.

Huerta-Cepas, Jaime, Damian Szklarczyk, Davide Heller, Ana Hernández-Plaza, Sofia K. Forslund, Helen Cook, Daniel R. Mende, et al. 2019. “eggNOG 5.0: A Hierarchical, Functionally and Phylogenetically Annotated Orthology Resource Based on 5090 Organisms and 2502 Viruses.” Nucleic Acids Research 47 (D1): D309–14.

Jain, Chirag, Luis M. Rodriguez-R, Adam M. Phillippy, Konstantinos T. Konstantinidis, and Srinivas Aluru. 2018. “High Throughput ANI Analysis of 90K Prokaryotic Genomes Reveals Clear Species Boundaries.” Nature Communications 9 (1): 5114.

Kolmogorov, Mikhail, Jeffrey Yuan, Yu Lin, and Pavel A. Pevzner. 2019. “Assembly of Long, Error-Prone Reads Using Repeat Graphs.” Nature Biotechnology 37 (5): 540–46.

Krzywinski, Martin, Jacqueline Schein, Inanç Birol, Joseph Connors, Randy Gascoyne, Doug Horsman, Steven J. Jones, and Marco A. Marra. 2009. “Circos: An Information Aesthetic for Comparative Genomics.” Genome Research 19 (9): 1639–45.

Li, Heng, and Richard Durbin. 2010. “Fast and Accurate Long-Read Alignment with Burrows-Wheeler Transform.” Bioinformatics 26 (5): 589–95.

Martin, Marcel. 2011. “Cutadapt Removes Adapter Sequences from High-Throughput Sequencing Reads.” EMBnet.journal 17 (1): 10–12.

Mikheenko, Alla, Vladislav Saveliev, and Alexey Gurevich. 2016. “MetaQUAST: Evaluation of Metagenome Assemblies.” Bioinformatics 32 (7): 1088–90.

Milanese, Alessio, Daniel R. Mende, Lucas Paoli, Guillem Salazar, Hans-Joachim Ruscheweyh, Miguelangel Cuenca, Pascal Hingamp, et al. 2019. “Microbial Abundance, Activity and Population Genomic Profiling with mOTUs2.” Nature Communications 10 (1): 1014.

Nurk, Sergey, Anton Bankevich, Dmitry Antipov, Alexey Gurevich, Anton Korobeynikov, Alla Lapidus, Andrey Prjibelsky, et al. 2013. “Assembling Genomes and Mini-Metagenomes

from Highly Chimeric Reads." Lecture Notes in Computer Science. https://doi.org/10.1007/978-3-642-37195-0_13.
Shani, Noam, Dino Isolini, Daniel Marzohl, and Hélène Berthoud. 2021. "Evaluation of a New Culture Medium for the Enumeration and Isolation of Streptococcus Salivarius Subsp. Thermophilus from Cheese." Food Microbiology. https://doi.org/10.1016/j.fm.2020.103672.
Simão, Felipe A., Robert M. Waterhouse, Panagiotis Ioannidis, Evgenia V. Kriventseva, and Evgeny M. Zdobnov. 2015. "BUSCO: Assessing Genome Assembly and Annotation Completeness with Single-Copy Orthologs." Bioinformatics 31 (19): 3210–12.
Sjöqvist, C., L. F. Delgado Zambrano, J. Alneberg, and A. F. Andersson. n.d. "Revealing Ecologically Coherent Population Structure of Uncultivated Bacterioplankton with POGENOM." https://doi.org/10.1101/2020.03.25.999755.
Tatusova, Tatiana, Michael DiCuccio, Azat Badretdin, Vyacheslav Chetvernin, Eric P. Nawrocki, Leonid Zaslavsky, Alexandre Lomsadze, Kim D. Pruitt, Mark Borodovsky, and James Ostell. 2016. "NCBI Prokaryotic Genome Annotation Pipeline." Nucleic Acids Research. https://doi.org/10.1093/nar/gkw569.
Tonkin-Hill, Gerry, Neil MacAlasdair, Christopher Ruis, Aaron Weimann, Gal Horesh, John A. Lees, Rebecca A. Gladstone, et al. 2020. "Producing Polished Prokaryotic Pangenomes with the Panaroo Pipeline." Genome Biology 21 (1): 180.
Vaser, Robert, Ivan Sović, Niranjan Nagarajan, and Mile Šikić. 2017. "Fast and Accurate de Novo Genome Assembly from Long Uncorrected Reads." Genome Research. https://doi.org/10.1101/gr.214270.116.
Vingataramin, Laurie, and Eric H. Frost. 2015. "A Single Protocol for Extraction of gDNA from Bacteria and Yeast." BioTechniques 58 (3): 120–25.
